# Accuracy of Metallic-Sleeved Versus Sleeve-Free Surgical Guides in Static Computer-Assisted Implant Surgery: A Systematic Review and Meta-Analysis

**DOI:** 10.3390/jcm15207935

**Published:** 2026-10-14

**Authors:** Kais Hijazi Muwaquet, Maria Dolores Gómez-Adrián, Julián Espinosa-Giménez, Jose Juan Enciso-Ripoll, Francisco Javier Villanueva-Arregui, Tawfiq Hijazi Alsadi, Susana Muwaquet Rodriguez, Javier Barberá-Millán

**Affiliations:** 1 Oral Surgery Unit, Department of Dentistry, Faculty of Medicine and Health Sciences, Catholic University of Valencia San Vicente Mártir, C/Quevedo, 2, 46001 Valencia, Spain; 2 Doctoral School, Catholic University of Valencia San Vicente Mártir, Valencia, Spain; 3 Department of Orthodontics, Faculty of Medicine and Health Sciences, Catholic University of Valencia San Vicente Mártir, C/Quevedo, 2, 46001 Valencia, Spain; 4 Department of Restorative Dentistry and Endodontics, Faculty of Medicine and Health Sciences, Catholic University of Valencia San Vicente Mártir, C/Quevedo, 2, 46001 Valencia, Spain

**Keywords:** static computer-assisted implant surgery, guided implant surgery, metallic sleeve, sleeve-free surgical guide, dental implant accuracy, angular deviation, apical deviation, meta-analysis

## Abstract

**Background/Objectives**: Metallic sleeves are widely used in static computer-assisted implant surgery (sCAIS), whereas sleeve-free guides may simplify the workflow. This systematic review and meta-analysis compared metallic-sleeved and sleeve-free guides for angular, coronal/platform, and apical deviation. **Methods**: The protocol was registered in the Open Science Framework and the review followed PRISMA 2020. PubMed/MEDLINE, Scopus, Web of Science, Cochrane Library/CENTRAL, and Google Scholar were searched; reports published in any language were eligible for assessment. Methodological quality and risk of bias were assessed using QUIN for in vitro studies and RoB 2 for the randomized trial. Random-effects meta-analyses were complemented by sensitivity and leave-one-out analyses. **Results**: Eleven studies were included (10 in vitro and one randomized controlled trial). Nine studies contributed to quantitative synthesis overall. Sleeve-free guides showed lower angular deviation (MD = −0.61°; 95% CI: −1.12 to −0.10; *p* = 0.019) and apical deviation (MD = −0.27 mm; 95% CI: −0.50 to −0.05; *p* = 0.017), while no statistically significant difference was detected for coronal/platform deviation (MD = −0.03 mm; 95% CI: −0.16 to 0.11; *p* = 0.689). Heterogeneity was substantial to high, and the 95% prediction intervals for angular and apical deviation crossed zero, reflecting variability across the evaluated guided systems. Sensitivity and leave-one-out analyses supported the overall direction of the findings. **Conclusions**: Sleeve-free guides showed modest average advantages in angular and apical deviation, while no statistically significant difference was detected for coronal/platform deviation. Performance depended on guide design, manufacturing accuracy, support, site morphology, and surgical protocol. The SLEEVE Rule was proposed as a structured framework to improve standardisation and comparability in future research. Further well-designed clinical studies are required to determine the clinical relevance of these technical differences.

## 1. Introduction

Static computer-assisted implant surgery (sCAIS) has become an important component of contemporary implant dentistry because it enables the transfer of a prosthetically driven virtual plan to the surgical field. By integrating cone-beam computed tomography, surface scanning, digital wax-ups, implant planning software, and computer-aided manufacturing, sCAIS allows implant positions to be planned three-dimensionally according to anatomical limitations and the intended restoration [1,2,3,4]. This is clinically relevant because successful implant therapy depends not only on osseointegration, but also on prosthetic emergence, screw-access trajectory, peri-implant tissue stability, cleanability, aesthetic integration, and long-term restorative predictability [2,3,4,5].

Previous systematic reviews have shown that static guided protocols generally improve implant placement accuracy compared with freehand approaches, although discrepancies between planned and achieved positions remain unavoidable [4,5,6,7,8]. These are commonly expressed as angular deviation, coronal or platform deviation, apical deviation, vertical/depth deviation, or three-dimensional global deviation. Even small discrepancies may become clinically relevant near adjacent teeth, thin buccal plates, sinus cavities, or neurovascular structures [6,7,8,9]. Implant placement accuracy should therefore be interpreted as both a technical performance measure and a determinant of biological safety and prosthetic feasibility.

The final implant position reflects cumulative errors throughout the digital and surgical workflow. Potential sources include image acquisition, segmentation, merging of Digital Imaging and Communications in Medicine and standard tessellation language files, virtual planning, guide support and fabrication, guide fit, surgical access, sleeve-to-bone distance, free drilling distance, instrumentation, implant macro-design, and operator-related variables [4,7,10,11,12]. Consequently, guided-surgery accuracy depends on the interaction between planning, manufacturing, guide design, instrumentation, and clinical execution rather than on the template alone.

Conventional static guides commonly incorporate metallic sleeves to guide drills, reduction keys, or implant carriers during osteotomy preparation and implant placement. Although metallic sleeves provide a rigid guiding interface, they may reduce visibility and external irrigation, increase the vertical space required for instrumentation, complicate posterior access or treatment in patients with limited mouth opening, increase system-specific costs, and add clinical and laboratory steps [13,14,15,16]. These limitations are particularly relevant in posterior implant placement, immediate extraction sockets, reduced interarch space, and cases requiring direct visual control.

Metallic-sleeved systems may also add cumulative tolerance between the guide body, metallic sleeve, drill key or adaptor, and drill. Each interface may permit a small degree of movement, which can be amplified as the distance between the guiding structure and the implant platform increases [11,12,17,18,19]. This tolerance-stack concept is central to the question of whether reducing interfaces can simplify the workflow and improve the transfer of the planned trajectory without compromising guide stability or material integrity.

Alternative designs have been developed to address these limitations. Open-sleeve systems preserve part of the sleeve geometry while permitting lateral drill insertion, which may improve posterior access and reduce the interarch space required for guided drilling [20,21,22,23]. They should be distinguished from true sleeve-free or sleeveless systems, which eliminate the metallic sleeve and incorporate guidance directly into the printed or milled guide body or through alternative keyless mechanisms [13,14,15,16,17,18,19]. Open and sleeve-free configurations may share access advantages but differ in guide-hole tolerance, wear, mechanical behaviour, and final implant deviation.

High-resolution additive manufacturing, improved milling strategies, and system-specific keyless protocols have increased the clinical relevance of sleeve-free guided surgery. Nevertheless, eliminating the metallic sleeve introduces potential concerns, including guide-hole wear, polymer debris, resin deformation, dimensional changes related to printing orientation or post-processing, and the need for precise internal guide-hole calibration [13,18,24,25]. The central question is therefore whether metallic sleeves can be removed while preserving or improving accuracy within a safe and reproducible workflow.

**Figure 1:**
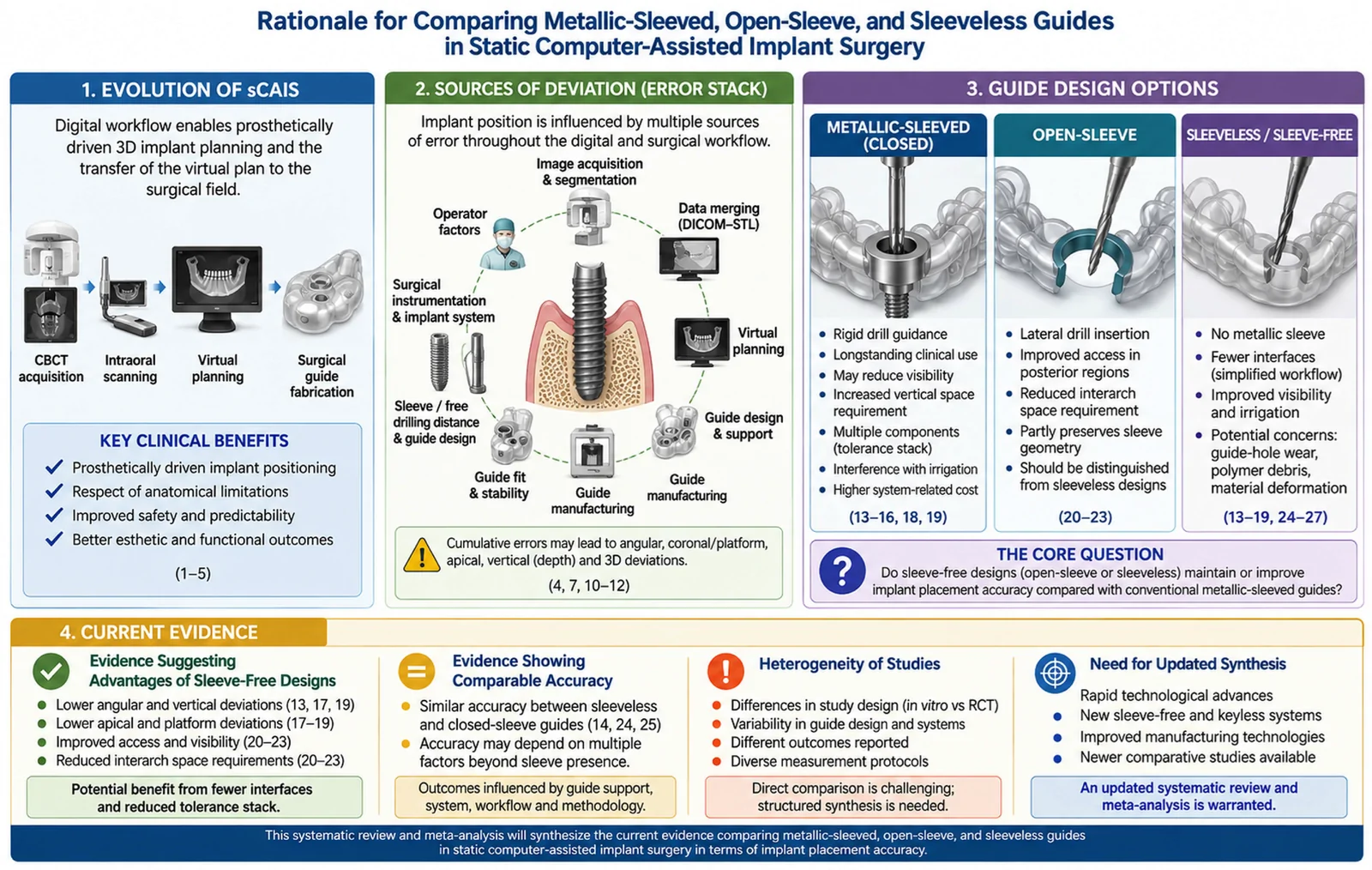
Rationale for comparing metallic-sleeved, open-sleeve, and sleeve-free surgical guides in static computer-assisted implant surgery. The diagram summarises the digital workflow, cumulative sources of deviation, principal guide-design concepts, and rationale for the present review [1,2,3,4,5,7,10,11,12,13,14,15,16,17,18,19,20,21,22,23,24,25,26,27].

The cumulative workflow factors and the main guide-design alternatives relevant to the present review are summarised in Figure 1.

Recent direct comparisons have reported either comparable or improved accuracy with sleeve-free designs, particularly for angular and apical deviation, while also showing that performance is influenced by guide geometry, drilling system, manufacturing method, sleeve-to-bone distance, and implant-site morphology [11,12,13,14,15,16,17,18,24,25,26,27]. Differences in the outcomes reported and in the conditions under which guides were tested complicate isolated interpretation and support an outcome-specific synthesis.

Previous reviews examined guided implant accuracy generally and templates with or without metallic sleeves specifically [28,29]. Esperouz et al. found no statistically significant differences in angular or horizontal deviation [29]. Since that review, additional comparative studies and contemporary workflows have become available [17,18,19,24,25,26,27]. The present review therefore evaluates angular, coronal/platform, and apical deviations separately in their original clinical units, examines healed and extraction-socket conditions, and tests the robustness of pooled estimates through sensitivity and leave-one-out analyses. The methodological implications emerging from this evidence are further synthesised in the Discussion through the SLEEVE Rule, a structured framework intended to support more standardised and comparable future research.

The predominance of controlled in vitro evidence is particularly relevant to this focused comparison. The same clinical implant site cannot be treated twice under identical anatomical, surgical, and prosthetic conditions, while patient-related factors such as bone density, ridge morphology, mouth opening, soft-tissue conditions, implant location, access, and visibility may obscure the isolated effect of guide design. Well-controlled in vitro comparisons therefore allow both concepts to be evaluated under standardised conditions and provide an important experimental basis for isolating the contribution of the metallic sleeve, guide-hole geometry, drilling system, and manufacturing protocol. Clinical validation remains necessary, but these models are informative for determining whether metallic sleeves are mechanically necessary for accurate transfer of the planned implant position.

This systematic review and meta-analysis aimed to determine whether metallic-sleeved and sleeve-free surgical guides differ in implant-placement accuracy, assessed separately through angular, coronal/platform, and apical deviation. For outcomes supported by sufficiently comparable data, between-design differences were quantified using outcome-specific meta-analyses, and the robustness of the pooled estimates was examined through sensitivity and leave-one-out analyses. Accordingly, the analysis examined whether the two guide concepts differed in any of the evaluated accuracy outcomes, without assigning superiority to either design.

## 2. Materials and Methods

### 2.1. Protocol and Reporting Framework

The protocol for this systematic review and meta-analysis was registered in the Open Science Framework (OSF; https://osf.io/tsqf7/) on 4 June 2026, prior to the final literature search performed on 5 June 2026. The subsequent stages of the review were conducted after protocol registration. This systematic review was designed and reported according to the Preferred Reporting Items for Systematic Reviews and Meta-Analyses (PRISMA) 2020 statement to ensure methodological transparency and reproducibility [30,31]. The completed PRISMA 2020 checklist is provided in Supplementary Materials (Table S1). No amendments were made after registration.

### 2.2. Review Design and PICOS Question

This study was designed as a systematic review of the literature with meta-analysis to compare the accuracy of static surgical guides with metallic sleeves versus surgical guides without metallic sleeves for dental implant placement.

The focused question was structured according to the PICOS framework and is presented in Table 1.

The PICOS question was: in dental implant placement using static guided surgery, do surgical guides without metallic sleeves present differences in accuracy compared with metallic-sleeved surgical guides in terms of angular, coronal/platform, and apical deviation?

### 2.3. Information Sources and Search Strategy

An electronic search was performed in PubMed/MEDLINE, Scopus, Web of Science, and the Cochrane Library/CENTRAL. Google Scholar was used as a complementary source, and the reference lists of included studies and relevant systematic reviews were manually screened to identify additional potentially eligible studies.

The search covered studies published between 2016 and 2026. The 2016–2026 period was prespecified to capture the most recent decade of evidence and thereby reflect contemporary sCAIS workflows, in which digital planning, CAD/CAM production, and additive manufacturing have become central to surgical-guide fabrication and accuracy [3,32]. The final search was performed on 5 June 2026. Reports published in any language were eligible for assessment. The search strategy combined terms related to dental implants, guided surgery, surgical guides, metallic sleeves, sleeveless/sleeve-free designs, and implant placement accuracy. The PubMed/MEDLINE strategy was adapted to the syntax of the remaining databases with the aid of the Polyglot Search Translator (Systematic Review Accelerator, Institute for Evidence-Based Healthcare, Bond University) [33], and the translated strategies were manually checked before execution. The search strategies were developed and conducted by K.H.M. and checked by J.B.-M. The complete search strategy is shown in Table 2.

### 2.4. Eligibility Criteria

The eligibility criteria were established before study selection and are summarised in Table 3.

For the purposes of this review, sleeve-free systems were defined as static surgical guides in which implant guidance was performed without a metallic guiding sleeve. Eligibility therefore required a direct within-study comparison between a guide without a metallic sleeve and a metallic-sleeved guide, irrespective of the specific sleeve-free implementation.

Previous systematic reviews were retained for background and discussion but were not included as primary studies in the qualitative or quantitative synthesis.

### 2.5. Study Selection Process

All records identified through the search strategy were collected using Zotero 7.0.30, and duplicates were removed. Titles and abstracts were screened by K.H.M. and checked by S.M.R. Potentially eligible studies were retrieved for full-text evaluation.

During full-text assessment, each article was evaluated to confirm whether it directly compared metallic-sleeved and sleeveless/sleeve-free guide designs and whether the reported data were suitable for qualitative extraction or quantitative synthesis. Full-text eligibility was independently assessed by K.H.M. and S.M.R., with disagreements resolved by consensus.

Inter-examiner agreement during the full-text eligibility assessment was evaluated using Cohen’s Kappa coefficient. Based on the independent classification of the 47 full-text reports by K.H.M. and S.M.R., the Cohen’s Kappa value was κ = 0.827, indicating strong agreement.

### 2.6. Data Extraction

Data were extracted using a standardised spreadsheet designed before the analysis. For each included study, the following data were recorded: internal study ID, author and year, title, study design, type of sample or population, number of implants or implant sites, type of guide support, description of the metallic-sleeved group, description of the sleeveless/sleeve-free group, presence of a direct comparison, measurement method, quantitative accuracy outcomes, units of measurement, study conclusion, inclusion decision for qualitative synthesis, inclusion decision for the main meta-analysis or sensitivity analysis, and methodological notes. Data extraction was performed by K.H.M. and verified by S.M.R. and M.D.G.-A. Study authors were not contacted for additional or missing data.

The full data extraction structure is summarised in Table 4.

For quantitative synthesis, means, standard deviations, and sample sizes were extracted for the metallic-sleeved and sleeveless/sleeve-free groups whenever available. Angular deviation was extracted in degrees, whereas coronal/platform and apical deviations were extracted in millimetres.

Studies relevant to the focused question but not providing directly comparable data for meta-analysis were included in the qualitative synthesis.

### 2.7. Outcomes

The primary outcome was angular deviation, expressed in degrees, between the planned implant axis and the final implant axis.

The secondary outcomes were:

1. coronal/platform deviation, expressed in millimetres;
2. apical deviation, expressed in millimetres;
3. three-dimensional/global deviation, when reported;
4. additional outcomes such as vertical deviation, horizontal deviation, guide fit, guide wear, surgical time, irrigation, or temperature, when available.

For meta-analysis, the most comparable outcomes across studies were prioritised: angular deviation, coronal/platform deviation, and apical deviation.

### 2.8. Methodological Quality and Risk-of-Bias Assessment

The methodological quality of in vitro studies was assessed using the Quality Assessment Tool for In Vitro Studies (QUIN) [34]. The QUIN tool evaluates 12 domains: clearly stated aims/objectives, sample size calculation, sampling technique/sample preparation, comparison group, methodology, operator details, randomization, outcome measurement method, outcome assessor details, blinding, statistical analysis, and presentation of results. Each domain was scored as 2 when adequately specified, 1 when partially or inadequately specified, and 0 when not specified. The total score was converted into a percentage. Studies scoring > 70% were classified as low risk of bias, studies scoring 50–70% as medium risk of bias, and studies scoring < 50% as high risk of bias. Methodological quality and risk-of-bias assessments were performed by K.H.M. and verified by T.H.A. and J.B.-M.

The randomized controlled trial by Tallarico et al. was assessed separately using the revised Cochrane Risk-of-Bias Tool for Randomized Trials (RoB 2) [35].

Additionally, certainty of evidence was assessed separately for the in vitro and clinical evidence using an adapted GRADE approach, considering risk of bias, inconsistency, indirectness, and imprecision. QUIN assessments informed the risk-of-bias judgements for the in vitro evidence, whereas RoB 2 informed the corresponding judgement for the randomized clinical trial. For the in vitro evidence, certainty was assessed in relation to comparative technical accuracy under controlled experimental conditions, while clinical evidence was evaluated separately. The complete setting-specific assessment is presented in Supplementary Table S2.

### 2.9. Data Preparation for Meta-Analysis

For each outcome, the mean, standard deviation, and sample size were extracted for both groups: metallic-sleeved guides and sleeveless/sleeve-free guides.

The effect size was calculated as a mean difference using the following direction:

*Sleeveless/sleeve-free* − *metallic-sleeved*

Therefore, negative values indicated lower deviation in the sleeveless/sleeve-free group, positive values indicated lower deviation in the metallic-sleeved group, and values close to zero indicated similar accuracy between both guide designs.

When a study reported clinically or methodologically distinct subgroups, such as healed sites and extraction sockets, these were treated as separate comparisons when the data represented different clinical conditions and could be extracted independently.

Sample sizes entered into the meta-analysis corresponded to the implant- or site-level observations reported by the primary studies. Where multiple observations originated from the same higher-level experimental unit (model, cast, or patient), these sample sizes therefore did not necessarily represent independent experimental units. The hierarchical structure of studies contributing to quantitative synthesis is summarised in Supplementary Table S4.

The study by Ballesteros et al. reported data as medians and interquartile ranges and was therefore excluded from the primary meta-analysis. For the sensitivity analysis, the reported median was used as an approximation of the mean, and the standard deviation was estimated using the approximation:

*SD* ≈ *IQR*/1.35

Because Raabe et al. and Dulla et al. were conducted by the same research group and shared closely related experimental characteristics, including standardised partially edentulous maxillary models, healed and extraction-socket morphologies, manufacturer’s sleeve versus sleeveless guide-hole comparisons, and similar digital planning and measurement workflows, they were not pooled simultaneously in the primary meta-analysis to minimise the risk of non-independent data contribution. The study by Raabe et al. was selected for the primary analysis because it provided the broader comparison for the present review question, whereas the study by Dulla et al. was used in a substitution sensitivity analysis.

### 2.10. Quantitative Synthesis and Statistical Analysis

Meta-analyses were performed for outcomes with sufficiently comparable data: angular deviation, coronal/platform deviation, and apical deviation. Since all outcomes were continuous and expressed in the same units within each outcome category, the mean difference was used as the effect measure.

Random-effects meta-analyses were fitted using inverse-variance weighting. Between-study variance (τ^2^) was estimated using restricted maximum likelihood (REML). Pooled mean differences, 95% confidence intervals, and two-sided *p*-values were calculated using standard normal (z-based) inference.

Statistical heterogeneity was assessed using τ^2^, the I^2^ statistic, and Cochran’s Q test. Ninety-five percent prediction intervals were additionally calculated from the fitted random-effects models. Given the limited number of comparisons within each outcome, heterogeneity estimates and prediction intervals were interpreted cautiously. The interpretation of results considered both statistical significance and the clinical direction and magnitude of the effect.

The statistical analysis was performed using R version 4.6.0 in RStudio Desktop. Meta-analyses were conducted using the metafor package version 5.0-1 [36], and tables were exported using openxlsx version 4.2.8.1.

### 2.11. Sensitivity Analyses

Sensitivity analyses were performed to evaluate the robustness of the main findings.

First, the study by Ballesteros et al. was added to the angular, coronal/platform, and apical deviation analyses after using the reported medians as approximations of the means and estimating standard deviations from the interquartile ranges.

Second, the study by Dulla et al. was used to replace the study by Raabe et al. in the angular, coronal/platform, and apical deviation analyses to assess whether possible methodological overlap affected the pooled estimates.

Third, leave-one-out analyses were performed by repeating each meta-analysis after removing one comparison at a time. This analysis was used to identify potentially influential studies or subgroups affecting the pooled estimate or heterogeneity.

Fourth, because the evidence base comprised predominantly in vitro studies together with one randomized clinical trial, an additional study-setting sensitivity analysis was performed by excluding the clinical trial by Tallarico et al. from the angular and coronal/platform meta-analyses. The apical meta-analysis was already based exclusively on in vitro studies and therefore required no additional study-setting sensitivity analysis.

Finally, an additional study-level sensitivity analysis was performed for Galli et al. by combining the healed-site and extraction-socket data within each guide group so that the study contributed a single comparison per outcome. This analysis was used to assess whether entering the two site conditions separately influenced the pooled estimates.

Because several studies included multiple implant/site observations nested within the same model, cast, or patient, potential within-cluster dependence was additionally examined. Cochrane guidance recommends direct meta-analysis of appropriately cluster-adjusted estimates and standard errors when available and approximate adjustment using cluster size and an intracluster correlation coefficient (ICC) when such information is unavailable [37]. The published primary studies did not provide study-specific ICCs or sufficiently detailed cluster-adjusted summary statistics from which exact adjusted variances could be reconstructed. Sampling variances were therefore inflated using the design effect, DE = 1 + (m − 1)ρ, where m was the mean number of observations per higher-level experimental unit and ρ the assumed ICC. Analyses were repeated for ICC values of 0, 0.10, 0.25, 0.50, 0.75, and 1.00. A further conservative analysis combined the healed- and extraction-site data from Galli et al. into a single study-level contribution before applying the same adjustment. Complete assumptions and results are provided in Supplementary Tables S4 and S5.

### 2.12. Assessment of Publication Bias

Publication bias was not formally assessed using funnel plots or statistical asymmetry tests because each meta-analysis included fewer than 10 comparisons. Under these conditions, funnel plot interpretation and asymmetry tests are considered unreliable.

## 3. Results

### 3.1. Study Selection

**Figure 2:**
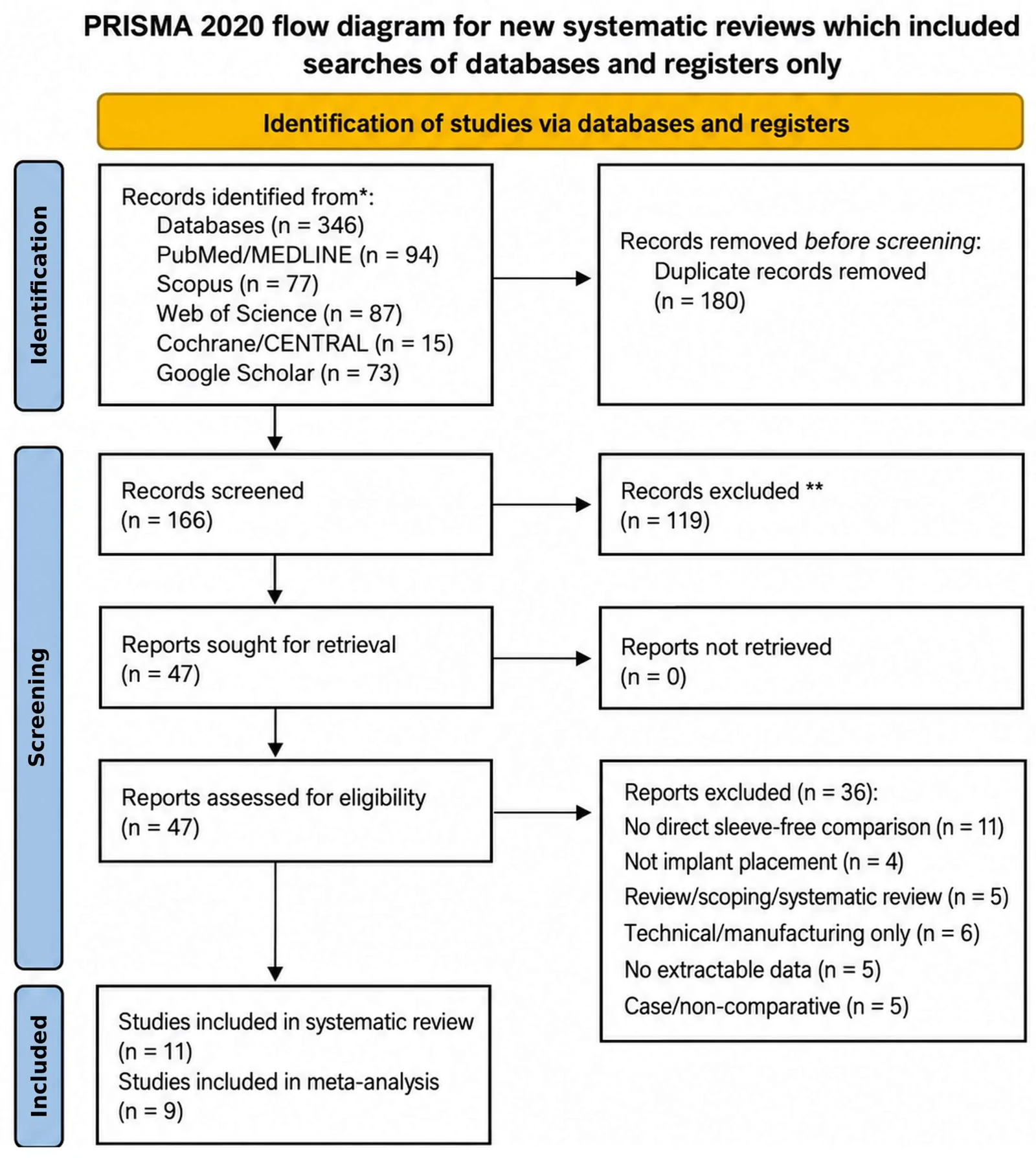
PRISMA 2020 flow diagram of study selection. The diagram shows record identification, duplicate removal, screening, full-text assessment, reasons for exclusion, and inclusion in the qualitative and quantitative syntheses. * The number of records identified is reported separately for each database searched. ** Records excluded during title and abstract screening (*n* = 119).

The study selection process is presented in Figure 2. A total of 346 records were identified through database and complementary searching: PubMed/MEDLINE (*n* = 94), Scopus (*n* = 77), Web of Science (*n* = 87), Cochrane/CENTRAL (*n* = 15), and Google Scholar (*n* = 73). After removal of 180 duplicate records, 166 records were screened by title and abstract. Of these, 119 records were excluded, and 47 full-text reports were assessed for eligibility. No reports were unavailable for retrieval.

After full-text assessment, 36 reports were excluded: 11 because they did not provide a direct sleeve-free comparison, 4 because they were not related to implant placement, 5 because they were review/scoping/systematic review articles, 6 because they were technical or manufacturing-only studies, 5 because they did not provide extractable data, and 5 because they were case reports or non-comparative studies. Finally, 11 studies were included in the systematic review, and 9 studies contributed to quantitative synthesis, including primary or sensitivity meta-analyses.

A study-level list of the 36 full-text reports excluded after eligibility assessment, together with the specific reason for exclusion of each report, is provided in Supplementary Table S3.

### 3.2. Characteristics and Quantitative Inclusion of Studies

The 11 studies included in the systematic review consisted of 10 in vitro studies and 1 randomized controlled trial. The principal study-level characteristics and comparative guided-surgery protocols are summarised in Table 5.

The quantitative synthesis was structured according to the three main outcomes defined in the review protocol: angular deviation, coronal/platform deviation, and apical deviation. Mean differences were calculated as sleeve-free minus sleeved; therefore, negative values indicate lower deviation in the sleeve-free group. Random-effects models were used for all meta-analyses because methodological heterogeneity was expected across studies, including differences in study design, guide system, model type, measurement method, implant site morphology, and manufacturing protocol.

The primary meta-analysis included eight comparisons for angular deviation, seven comparisons for coronal/platform deviation, and six comparisons for apical deviation. Ballesteros et al. [26] was excluded from the primary analysis because its original data were reported as medians and interquartile ranges; it was included only in sensitivity analyses after approximate conversion to means and standard deviations. Dulla et al. [17] was not pooled together with Raabe et al. [18] because of possible methodological overlap and was therefore used only in a replacement sensitivity analysis. The outcome-specific quantitative data and analytical inclusion of the included studies are summarised in Table 6.

### 3.3. Qualitative Synthesis of Included Studies

The qualitative synthesis included all 11 studies retained in the systematic review.

In the randomized controlled trial by Tallarico et al. [13], surgical templates without metallic sleeves showed lower angular and vertical deviations than conventional templates with metallic sleeves, while no implant failures or surgical complications were reported. Within the sleeve-free group, closed sleeve-designed templates were more accurate than open designs, particularly for angular and horizontal deviations.

Among the in vitro studies, several investigations reported findings favouring sleeve-free or sleeveless guide designs. Dulla et al. [17] found that sleeveless guide-hole designs produced significantly lower apical and angular deviations than manufacturer’s sleeve designs. The study also reported lower deviations in healed ridges than in fresh extraction sockets. Similarly, Raabe et al. [18] reported smaller angular, crestal, and apical deviations with sleeveless guide-hole designs compared with manufacturer’s sleeve designs and freehand placement. Healed ridges also showed lower deviations than extraction sockets, while guide-hole deterioration was greater at sleeveless sites.

Hang and Guentsch [12] directly evaluated sleeved and sleeveless guides at different sleeve-to-bone distances. Sleeveless guides showed significantly lower angular, platform, and apical deviations than sleeved guides. Angular deviation increased as the distance from the guide to the implant platform increased.

Galli et al. [14] compared a novel sleeveless guide with a conventional closed-sleeve guide and a freehand approach in both healed sites and fresh extraction sockets. In extraction socket sites, the sleeveless guide showed lower angular deviation than the freehand group and numerically lower angular deviation than the conventional guide, although the conventional closed-sleeve guide showed lower coronal horizontal deviation than the sleeveless guide. In healed sites, the sleeveless guide demonstrated significantly lower angular deviation than the conventional closed-sleeve guide and freehand placement.

Ballesteros et al. [26] evaluated additive manufactured guides with metal sleeves, additive manufactured guides without metal sleeves, and subtractive manufactured guides without metal sleeves. Non-sleeved guides showed lower crestal and apical deviations than metal-sleeved printed guides. Differences were also observed between the two non-sleeved groups: milled non-sleeved guides produced the lowest crestal and apical deviations, whereas printed non-sleeved guides showed the lowest angular deviation.

Other studies reported comparable rather than clearly superior performance of sleeve-free designs. Chung et al. [15] found that closed metal sleeve and closed non-metal sleeve templates produced similar accuracy, while open non-metal sleeve templates tended to show larger coronal, apical, and angular deviations. Oh et al. [24] reported no significant difference in guide weight before and after implant placement and generally comparable angular deviations between metal sleeve-free and metal sleeve-incorporated 3D-printed guides, with one mesiodistal angular parameter favouring the sleeve-free group. Aboul Azayem et al. [16] also found slightly lower angular deviation in the metal-free group for both premolar and molar implants, although the differences were not statistically significant.

Sittikornpaiboon et al. [11] compared five different drilling system configurations and showed that the overall accuracy of sCAIS was influenced by the drilling system design. The integrated sleeve-on-drill system without a metal sleeve did not differ significantly from the equivalent system with a metal sleeve, but both differed from other guided systems. Adams et al. [25] similarly reported that digitally designed guides without metal sleeves demonstrated similar accuracy and higher precision compared with conventional metal-sleeved guides. Because Adams et al. reported mainly directional and signed deviations rather than directly comparable global coronal, apical, and angular outcomes, the study was retained in the qualitative synthesis but was not included in the main meta-analysis.

### 3.4. Methodological Quality and Risk of Bias

**Figure 3:**
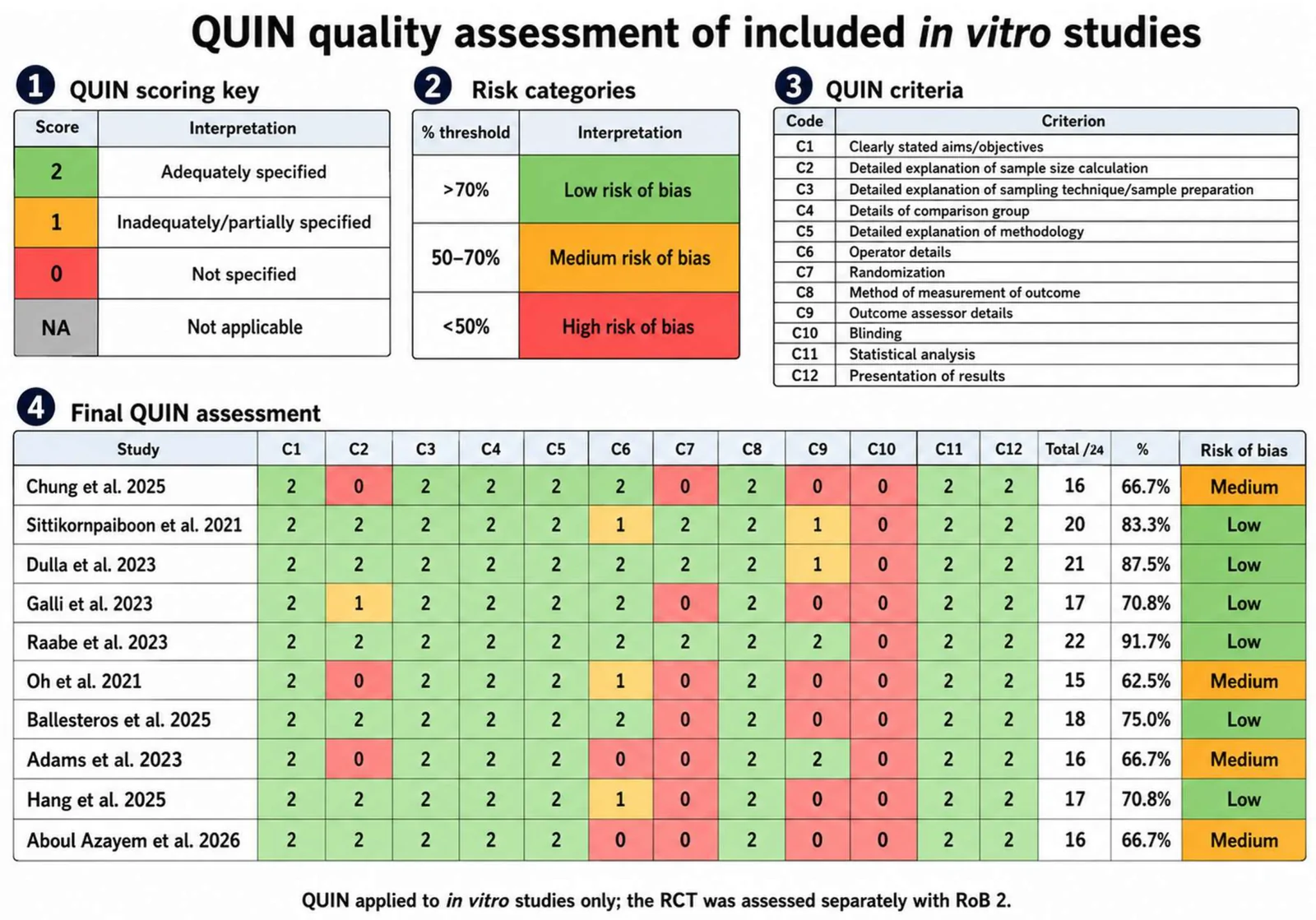
QUIN assessment of the included in vitro studies. Domain-level scores and overall methodological-quality classifications are shown for the 10 included in vitro studies [11,12,14,15,16,17,18,24,25,26] according to the QUIN tool [34].

The methodological quality of the 10 in vitro studies was assessed using the QUIN tool. Six studies were classified as having low risk of bias, while four studies were classified as having medium risk of bias. No in vitro study was classified as high risk of bias. The studies classified as low risk of bias were Sittikornpaiboon et al. [11], Dulla et al. [17], Galli et al. [14], Raabe et al. [18], Ballesteros et al. [26], and Hang et al. [12]. The studies classified as medium risk of bias were Chung et al. [15], Oh et al. [24], Adams et al. [25], and Aboul Azayem et al. [16]. The full QUIN assessment is summarised in Figure 3.

The most frequently unmet QUIN domains were blinding, outcome assessor reporting, and randomization. Several studies used standardised models, controlled digital workflows, and software-based deviation measurements; however, explicit blinding of outcome assessment was not reported in any in vitro study. Outcome assessor details were frequently absent or incomplete, although some studies used objective digital superimposition procedures that reduced manual measurement subjectivity.

**Figure 4:**
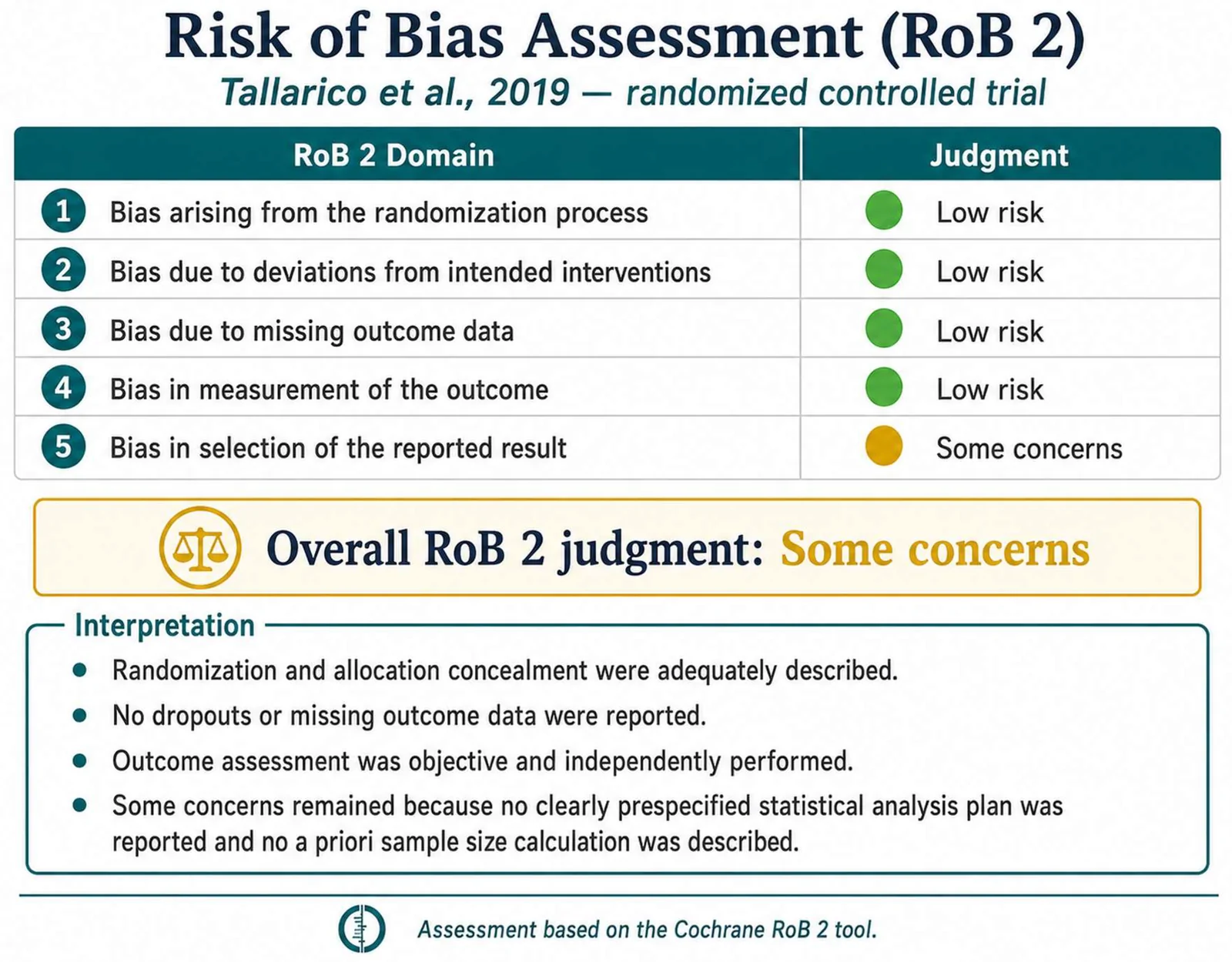
RoB 2 assessment of the randomised controlled trial by Tallarico et al. [13]. Domain-level judgements and the overall risk-of-bias judgement are presented according to the RoB 2 tool [35].

The randomized controlled trial by Tallarico et al. [13] was assessed using RoB 2. Four domains were judged as low risk of bias: bias arising from the randomization process, bias due to deviations from intended interventions, bias due to missing outcome data, and bias in measurement of the outcome. Domain 5, bias in selection of the reported result, was judged as having some concerns because no clearly pre-specified statistical analysis plan was available and no a priori sample size calculation was reported. The overall RoB 2 judgment was therefore classified as having some concerns (Figure 4). Setting-specific certainty assessments for the in vitro and clinical evidence are presented in Supplementary Table S2.

### 3.5. Primary Meta-Analysis

The primary random-effects meta-analysis evaluated angular, coronal/platform, and apical deviation separately. Mean differences were calculated as sleeve-free minus sleeved; negative values therefore indicated lower deviation with sleeve-free guides. The I^2^ values represent heterogeneity within each outcome rather than the pooling of different deviation parameters.

**Figure 5:**
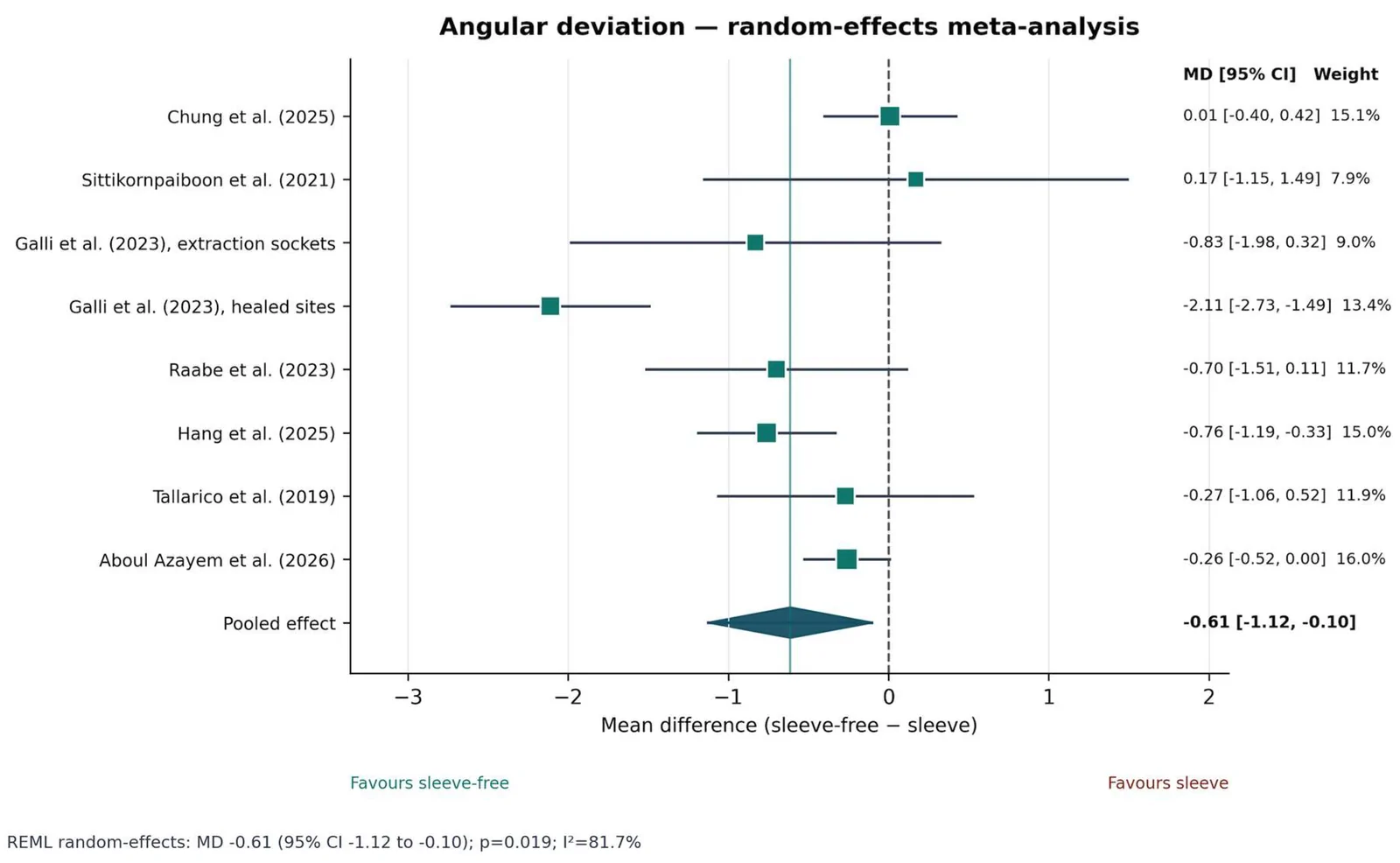
Forest plot of angular deviation [11,12,13,14,15,16,18]. Random-effects mean differences were calculated as sleeve-free minus metallic-sleeved; negative values favour sleeve-free guides. The pooled estimate favoured sleeve-free guides (MD = −0.61°; 95% CI, −1.12 to −0.10; *p* = 0.019; I^2^ = 81.7%).

For angular deviation, eight comparisons were included in the primary analysis. The pooled estimate showed lower angular deviation with sleeve-free guides (MD = −0.61°; 95% CI: −1.12 to −0.10; *p* = 0.019), with substantial heterogeneity (I^2^ = 81.7%). Most individual comparisons were located on the sleeve-free side of the forest plot. The largest individual effects were observed for Galli et al. [14] at healed sites and Hang and Guentsch [12], whereas Chung et al. [15] showed virtually no difference and Sittikornpaiboon et al. [11] slightly favoured the sleeved group. The angular-deviation forest plot is shown in Figure 5.

**Figure 6:**
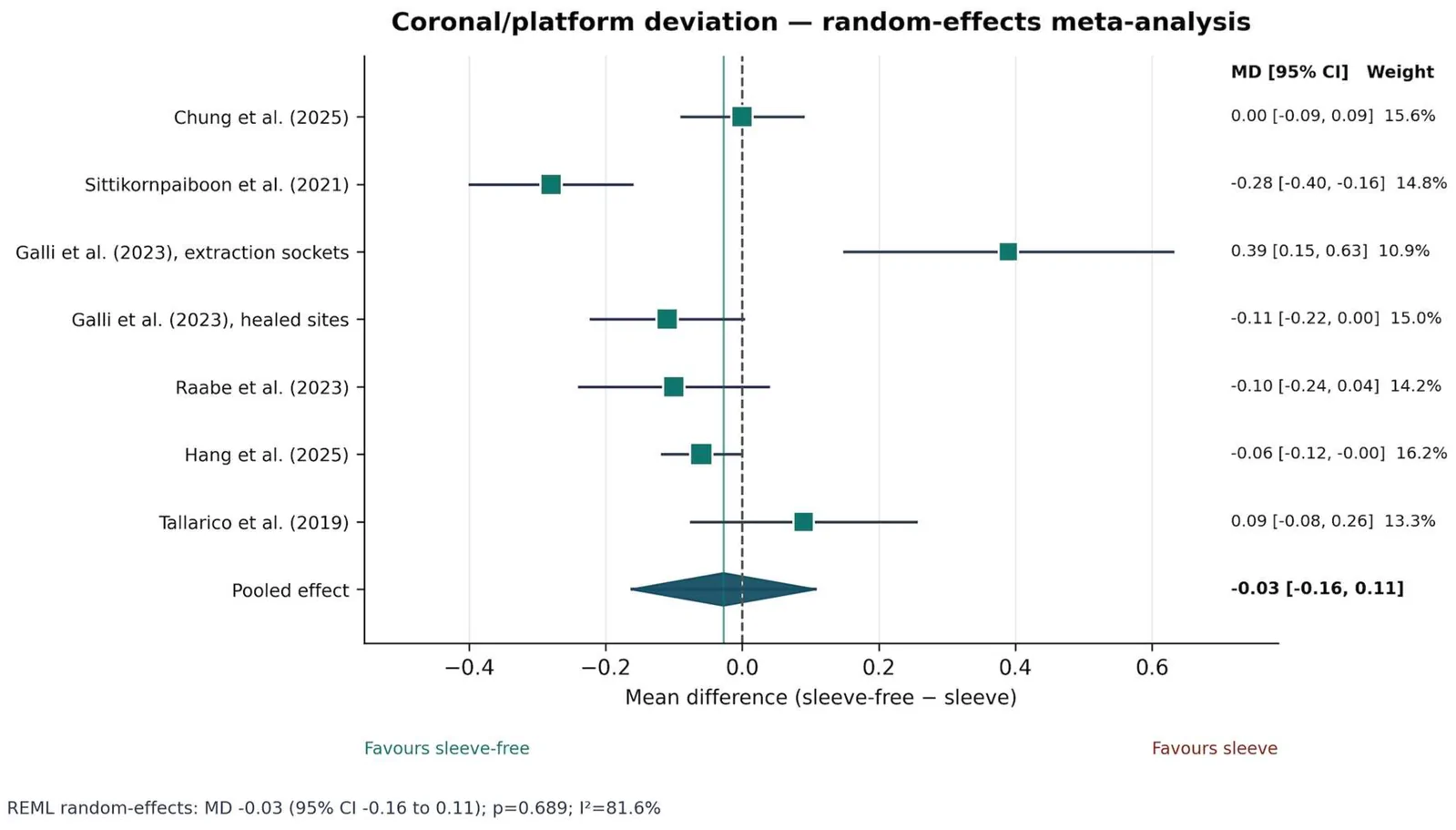
Forest plot of coronal/platform deviation [11,12,13,14,15,18]. Random-effects mean differences were calculated as sleeve-free minus metallic-sleeved; negative values favour sleeve-free guides. The pooled estimate showed no significant difference between guide designs (MD = −0.03 mm; 95% CI, −0.16 to 0.11; *p* = 0.689; I^2^ = 81.6%).

For coronal/platform deviation, seven comparisons were included. The pooled estimate was close to zero and was not statistically significant (MD = −0.03 mm; 95% CI: −0.16 to 0.11; *p* = 0.689). Individual study effects varied in direction, with some comparisons favouring sleeve-free guides and others favouring sleeved guides. Substantial heterogeneity was observed (I^2^ = 81.6%). The coronal/platform-deviation forest plot is shown in Figure 6.

**Figure 7:**
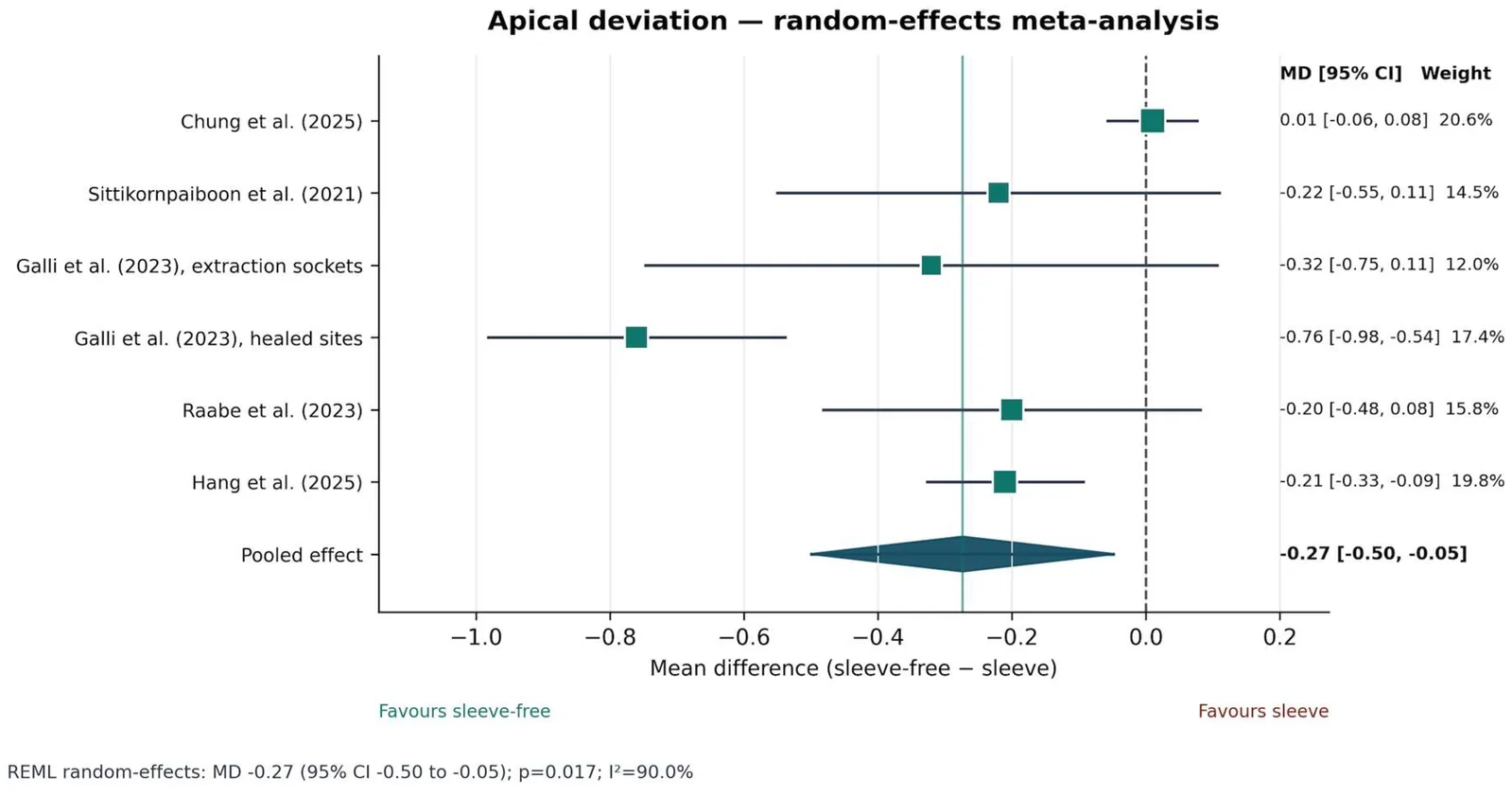
Forest plot of apical deviation [11,12,14,15,18]. Random-effects mean differences were calculated as sleeve-free minus metallic-sleeved; negative values favour sleeve-free guides. The pooled estimate favoured sleeve-free guides (MD = −0.27 mm; 95% CI, −0.50 to −0.05; *p* = 0.017; I^2^ = 90.0%).

For apical deviation, six comparisons were included. The pooled estimate showed lower apical deviation with sleeve-free guides (MD = −0.27 mm; 95% CI: −0.50 to −0.05; *p* = 0.017). Most individual comparisons favoured sleeve-free guides, with the largest effect observed for the healed-site subgroup of Galli et al. [14]. Heterogeneity was high (I^2^ = 90.0%). The apical-deviation forest plot is shown in Figure 7.

The primary meta-analysis results are summarised in Table 7.

### 3.6. Sensitivity Analyses

Sensitivity analyses supported the direction of the primary findings. Adding Ballesteros et al. [26] after conversion of interquartile ranges to estimated standard deviations retained significant effects for angular deviation (MD = −0.79°; 95% CI: −1.37 to −0.22; *p* = 0.007) and apical deviation (MD = −0.30 mm; 95% CI: −0.51 to −0.10; *p* = 0.004), while coronal/platform deviation remained non-significant (MD = −0.06 mm; 95% CI: −0.20 to 0.08; *p* = 0.388). Heterogeneity remained substantial to high.

Replacing Raabe et al. [18] with Dulla et al. [17] produced results almost identical to the primary analysis: angular deviation remained significant (MD = −0.62°; 95% CI: −1.13 to −0.10; *p* = 0.019), apical deviation remained significant (MD = −0.28 mm; 95% CI: −0.51 to −0.06; *p* = 0.015), and coronal/platform deviation remained non-significant (MD = −0.03 mm; 95% CI: −0.16 to 0.11; *p* = 0.706). These estimates were consistent with the primary analyses.

**Figure 8:**
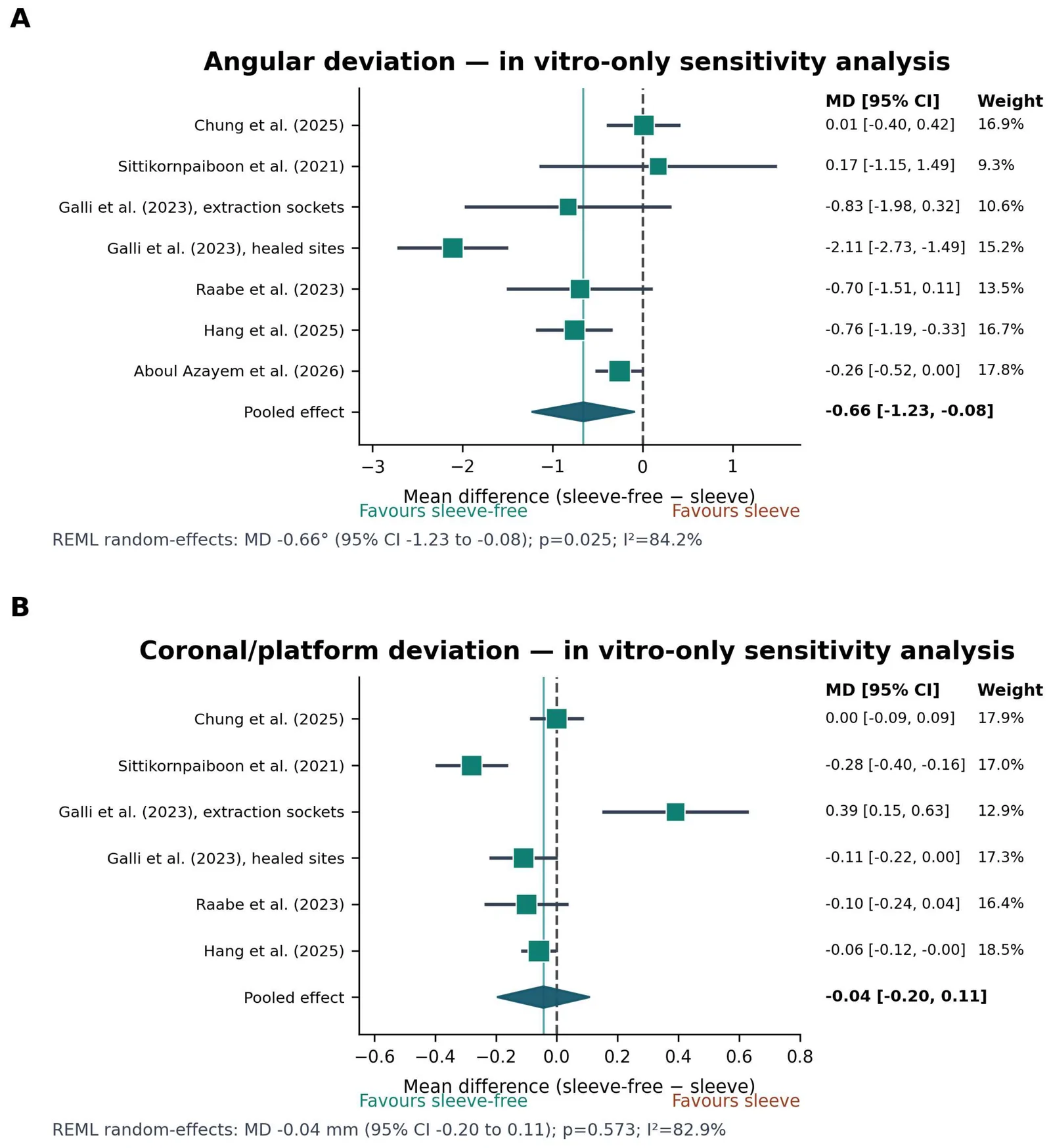
Study-setting sensitivity analyses excluding the randomized clinical trial by Tallarico et al. [13]. (**A**) Angular deviation [11,12,14,15,16,18]. The pooled estimate remained significantly in favour of sleeve-free guides (MD = −0.66°; 95% CI, −1.23 to −0.08; *p* = 0.025; I^2^ = 84.2%). (**B**) Coronal/platform deviation [11,12,14,15,18]. The pooled estimate remained non-significant (MD = −0.04 mm; 95% CI, −0.20 to 0.11; *p* = 0.574; I^2^ = 82.9%). Random-effects mean differences were calculated as sleeve-free minus metallic-sleeved; negative values favour sleeve-free guides.

An additional study-setting sensitivity analysis excluding the randomized clinical trial by Tallarico et al. [13] produced findings consistent with the primary analyses. For angular deviation, the pooled effect remained statistically significant in favour of sleeve-free guides (MD = −0.66°; 95% CI: −1.23 to −0.08; *p* = 0.025; I^2^ = 84.2%). For coronal/platform deviation, the pooled estimate remained close to zero and non-significant (MD = −0.04 mm; 95% CI: −0.20 to 0.11; *p* = 0.574; I^2^ = 82.9%). The apical meta-analysis already consisted entirely of in vitro evidence. The corresponding in vitro-only sensitivity analyses are shown in Figure 8.

Combining the healed-site and extraction-socket data from Galli et al. into a single study-level comparison yielded similar pooled estimates. Angular deviation remained significantly lower with sleeve-free guides (MD = −0.51°; 95% CI: −0.93 to −0.09; *p* = 0.018), coronal/platform deviation remained non-significant (MD = −0.04 mm; 95% CI: −0.15 to 0.07; *p* = 0.426), and apical deviation remained significantly lower with sleeve-free guides (MD = −0.22 mm; 95% CI: −0.42 to −0.03; *p* = 0.025).

Adjustment for potential within-cluster dependence did not alter the interpretation of any outcome when the primary meta-analytic structure was retained, even at ICC = 1.00 (Supplementary Table S5). In the more conservative analysis combining the two Galli et al. site conditions before clustering adjustment, angular deviation remained significantly lower with sleeve-free guides at ICC = 1.00 (MD = −0.42°; 95% CI, −0.75 to −0.08; *p* = 0.015), whereas coronal/platform deviation remained non-significant (MD = −0.07 mm; 95% CI, −0.16 to 0.03; *p* = 0.162). For apical deviation, the pooled estimate remained in favour of sleeve-free guidance throughout the complete ICC range and remained statistically significant through ICC = 0.50 (MD = −0.17 mm; 95% CI, −0.34 to −0.004; *p* = 0.044); under stronger assumed correlations, the confidence interval included the null.

The sensitivity analyses are summarised in Table 8.

### 3.7. Leave-One-Out Analysis

Leave-one-out analysis showed that the angular effect generally remained in favour of sleeve-free guides. Omitting the healed-site subgroup of Galli et al. [14] reduced the pooled effect from −0.61° to −0.36° and heterogeneity from 81.7% to 32.4%.

For coronal/platform deviation, no individual omission changed the non-significant conclusion, and the pooled effect remained close to zero. Omitting the extraction-socket subgroup of Galli et al. [14] produced the largest reduction in heterogeneity, although substantial variability remained.

For apical deviation, the pooled effect remained in favour of sleeve-free guides after each omission. Excluding the healed-site subgroup of Galli et al. [14] reduced the mean difference from −0.27 mm to −0.14 mm and heterogeneity from 90.0% to 71.2%.

The leave-one-out results are summarised in Table 9.

### 3.8. Publication Bias

Publication bias was not formally assessed because fewer than 10 comparisons were included in each primary meta-analysis. Therefore, funnel plot asymmetry and regression-based tests for small-study effects were not considered appropriate. Consequently, small-study effects and publication bias cannot be ruled out.

## 4. Discussion

### 4.1. Principal Findings

This systematic review and meta-analysis found lower average angular and apical deviations with sleeve-free guides, while no statistically significant difference was detected for coronal/platform deviation. The primary effects were −0.61° for angular deviation and −0.27 mm for apical deviation, with a coronal/platform difference close to zero. Substantial to high heterogeneity indicates that the magnitude of these effects varies across systems and study contexts.

Guided implant surgery aims to transfer a prosthetically driven plan as accurately as possible. Although previous reviews have shown that sCAIS improves placement accuracy compared with freehand surgery, residual deviation remains unavoidable [1,4,5,6,7,8]. The present review adds a focused message: a metallic sleeve is not an automatic prerequisite for accurate placement, and sleeve-free designs may provide a modest trajectory-related advantage when the complete system is appropriately designed and manufactured.

The outcome pattern is clinically and mechanically coherent. At the coronal/platform level, the pooled effect remained close to zero, whereas lower angular and apical deviation may reflect improved control along the osteotomy axis. This distinction is more informative than interpreting guided accuracy as a single outcome.

### 4.2. Interpretation of Angular Deviation Findings

Angular deviation was the most consistently reported outcome and represents one of the most important parameters in guided implant surgery. The pooled analysis showed a statistically significant reduction of angular deviation in favour of sleeve-free guides. Although the mean difference of −0.61° may appear numerically modest, angular discrepancies can be amplified over the implant length and may contribute to larger apical positional errors, especially in longer implants or anatomically limited sites. However, the substantial heterogeneity and the influence of the Galli et al. healed-site subgroup indicate that the magnitude of this angular advantage is unlikely to be identical across all sleeve-free systems or site conditions.

The direction of the pooled effect was supported by several included studies. Hang and Guentsch reported significantly lower angular deviation for sleeveless guides compared with sleeved guides and also showed that angular deviation increased as the guide-to-platform distance increased [12]. This finding is particularly relevant because it supports the mechanical concept that small guidance errors can be magnified when the distance between the guide and the implant platform increases. Dulla et al. and Raabe et al. also reported lower angular deviations when sleeveless guide-hole designs were used compared with manufacturer’s sleeve designs, supporting the hypothesis that reducing guide-related interfaces may improve control of the drilling trajectory [17,18]. Galli et al. found that the sleeveless guide showed particularly favourable angular results at healed sites, while Tallarico et al. reported lower angular deviation in the clinical group treated with templates without metallic sleeves [13,14].

A plausible explanation is the reduction of cumulative tolerances. In conventional metallic-sleeved systems, the drilling trajectory may depend on several interfaces: the printed or milled guide body, the metallic sleeve, the drill key or adaptor when present, and the drill itself. Each interface may introduce a small degree of freedom, and these small tolerances may accumulate during osteotomy preparation. This is consistent with the rationale described by Sittikornpaiboon et al., who emphasized that different drilling system configurations can affect implant placement accuracy [11], and with the guide-hole design concepts described by Dulla et al. and Raabe et al. [17,18]. It is also consistent with evidence that drill play and lateral forces may influence final implant position in guided implantology [19].

However, this explanation should not be interpreted as proof that sleeve-free designs are inherently superior in all situations. Sleeve-free guides transfer guidance directly through the guide material or through a simplified keyless mechanism, and this makes their performance highly dependent on guide-hole calibration, material rigidity, printing or milling accuracy, post-processing, drill-to-guide fit, and resistance to guide-hole wear. Raabe et al. observed greater volumetric material reduction in sleeveless guide holes compared with manufacturer’s sleeve sites, indicating that material wear may represent a relevant limitation of some sleeve-free systems [18]. Therefore, the mechanical simplification of sleeve-free guides may reduce certain interface-related errors, but it also increases the importance of manufacturing control and material stability.

### 4.3. Interpretation of Coronal/Platform Deviation Findings

In contrast to angular deviation, coronal/platform deviation showed no statistically significant difference between metallic-sleeved and sleeve-free guides. The pooled mean difference was −0.03 mm, which is extremely small and unlikely to be clinically meaningful. The pooled analysis therefore did not detect a statistically significant between-design difference at the coronal/platform level.

This outcome may be influenced by factors that are not exclusively related to sleeve design. Coronal/platform accuracy is likely affected by guide support, guide seating, tooth support stability, guide fit, local anatomy, and the definition of the measurement point. Several included studies used tooth-supported models or partially edentulous scenarios, which may provide good initial guide stability. In such conditions, the presence or absence of a metallic sleeve may have less influence on the coronal entry point than on the angular trajectory or apical endpoint.

The inconsistency between studies also deserves attention. Some comparisons favoured sleeve-free guides, whereas others favoured sleeved guides. Galli et al. reported that the conventional closed-sleeve guide showed lower coronal horizontal deviation than the sleeveless guide at extraction socket sites, even though the sleeveless guide showed favourable angular performance [14]. Tallarico et al. also contributed to the direction favouring sleeved guides for some horizontal or coronal parameters, while Hang and Guentsch, Raabe et al., and Sittikornpaiboon et al. showed findings that were more favourable or comparable for sleeve-free designs [11,12,13,18]. This variability likely explains why the pooled effect remained close to zero.

Another important issue is outcome definition. Across studies, similar but not identical terms were used, including coronal deviation, platform deviation, crestal deviation, horizontal deviation, lateral deviation, or two-dimensional platform deviation. Although these outcomes are conceptually related, they may not be perfectly interchangeable. Differences in software, superimposition method, scan body use, CBCT-based measurement, STL-based measurement, and reference point definition may all contribute to heterogeneity. Therefore, the most defensible conclusion is not that sleeve-free guides improve coronal/platform accuracy, but rather that they do not appear to compromise it.

### 4.4. Interpretation of Apical Deviation Findings

Apical deviation was lower with sleeve-free guides, with a pooled mean difference of −0.27 mm. Although this magnitude is modest, it may be clinically meaningful because the implant apex is often closest to critical anatomical structures, including the mandibular canal, maxillary sinus, nasal floor, cortical plates, or adjacent roots. Even small improvements in apical accuracy may be relevant when implants are planned in anatomically constrained sites. Nevertheless, the high heterogeneity observed for this outcome means that the pooled value should be interpreted as an average estimate rather than as a predictable effect for all clinical scenarios.

The apical finding is also biomechanically coherent with the angular result. Angular deviation and apical deviation are geometrically linked: a small change in implant angulation can result in a larger displacement at the apex, particularly as implant length increases. Therefore, the simultaneous reduction in angular and apical deviations supports the hypothesis that sleeve-free designs may improve trajectory control rather than simply alter the coronal entry point.

The included studies support this interpretation. Dulla et al. found significantly lower apical deviation with sleeveless guide-hole designs compared with manufacturer’s sleeves [17]. Raabe et al. similarly reported smaller apical deviations with sleeveless guide-hole designs than with manufacturer’s sleeve designs and freehand placement [18]. Hang and Guentsch found lower three-dimensional apical deviation in sleeveless guides compared with sleeved guides [12]. Galli et al. also contributed substantially to the apical effect, particularly in healed sites [14].

Nevertheless, the high heterogeneity observed for apical deviation indicates that the magnitude of this benefit is not uniform. Implant site morphology appears to be especially important. Dulla et al. reported substantially greater deviations in fresh extraction sockets than in healed ridges, regardless of guide-hole design [17]. Raabe et al. also reported higher deviations in extraction sockets compared with healed ridges [18]. Similarly, Galli et al. showed that healed and extraction socket sites behaved differently, with the healed-site subgroup contributing strongly to the pooled apical effect [14]. These findings indicate that local anatomy and ridge morphology may modify the performance of both sleeved and sleeve-free guided systems.

### 4.5. Role of Heterogeneity and Methodological Variability

Substantial to high heterogeneity was observed across all three outcomes. This was expected because the included studies differed in design, sample type, guide support, implant system, drilling protocol, guide-hole design, manufacturing method, measurement method, and implant site morphology. Most studies were in vitro, but they did not use identical models or protocols. Some used maxillary models, others mandibular models; some evaluated healed ridges, others extraction sockets; some compared manufacturer’s sleeves with sleeveless guide holes, whereas others evaluated open-frame sleeveless systems, non-metal sleeve designs, or 3D-printed metal sleeve-free guides [11,12,13,14,15,16,17,18,24,25,26].

Contemporary sleeve-free systems apply the same core design principle—the elimination of the metallic guiding sleeve—through different guide geometries, materials, and instrumentation workflows. Such technological variation is expected in a meta-analysis of evolving guided-surgery systems and was addressed through outcome-specific random-effects models, sensitivity analyses, and leave-one-out analyses. Chung et al. showed that closed non-metal sleeve templates were comparable to closed metal sleeve templates, whereas open non-metal designs tended to show larger deviations [15]; Tallarico et al. likewise found greater accuracy for closed than open sleeve-free designs for some parameters [13]. Accordingly, the pooled estimates should be interpreted as comparisons between complete metallic-sleeved and sleeve-free guided systems across contemporary implementations, rather than as an isolated effect of the metallic sleeve; final accuracy also depends on the design and stability of the complete workflow. The observed heterogeneity therefore reflects variation in the magnitude of a common system-level comparison across contemporary workflows rather than inconsistency in the fundamental intervention contrast.

The leave-one-out analysis identified the healed-site subgroup of Galli et al. as the most influential comparison for angular and apical deviation [14]. Its omission reduced the angular MD from −0.61° to −0.36° and I^2^ from 81.7% to 32.4%; for apical deviation, the MD decreased from −0.27 mm to −0.14 mm and I^2^ from 90.0% to 71.2%. The direction of both effects remained in favour of sleeve-free guides, and the study-level sensitivity analysis combining the healed- and extraction-site data from Galli et al. retained statistically significant angular and apical pooled effects.

### 4.6. Robustness of the Findings

The sensitivity analyses strengthen the credibility of the main findings. The study by Ballesteros et al. was not included in the primary meta-analysis because its original data were reported as medians and interquartile ranges rather than means and standard deviations [26]. When this study was added after approximate conversion of interquartile ranges to estimated standard deviations, the pooled effects for angular and apical deviation remained statistically significant and even increased slightly. Coronal/platform deviation remained non-significant. This suggests that the exclusion of Ballesteros et al. from the primary analysis did not artificially generate the main findings.

The second sensitivity analysis addressed the potential non-independence between Raabe et al. and Dulla et al. [17,18]. These studies were conducted by closely related research groups and shared important experimental characteristics, including standardised partially edentulous maxillary models, healed and extraction-socket morphologies, manufacturer’s sleeve versus sleeveless guide-hole comparisons, and similar guided-surgery workflows [17,18]. For this reason, they were not pooled simultaneously in the primary analysis, to minimise the risk of non-independent data contribution. Replacing Raabe et al. with Dulla et al. produced results that were almost identical to the primary analysis for angular, coronal/platform, and apical deviation. This indicates that the conclusions were robust to the selection of either of these two closely related studies.

The study-setting sensitivity analysis provided an additional assessment of robustness. Excluding the only randomized clinical trial produced essentially the same interpretation as the primary analyses: angular deviation remained significantly lower with sleeve-free guides, whereas coronal/platform deviation remained non-significant. The apical analysis was already entirely based on in vitro studies. These findings indicate that the direction of the pooled results was not dependent on the inclusion of the clinical study, while the predominance of laboratory evidence remains important when considering clinical generalisability.

The additional clustering sensitivity analysis further supported the overall interpretation, indicating that the principal findings were not dependent on treating nested implant/site observations as fully independent.

**Figure 9:**
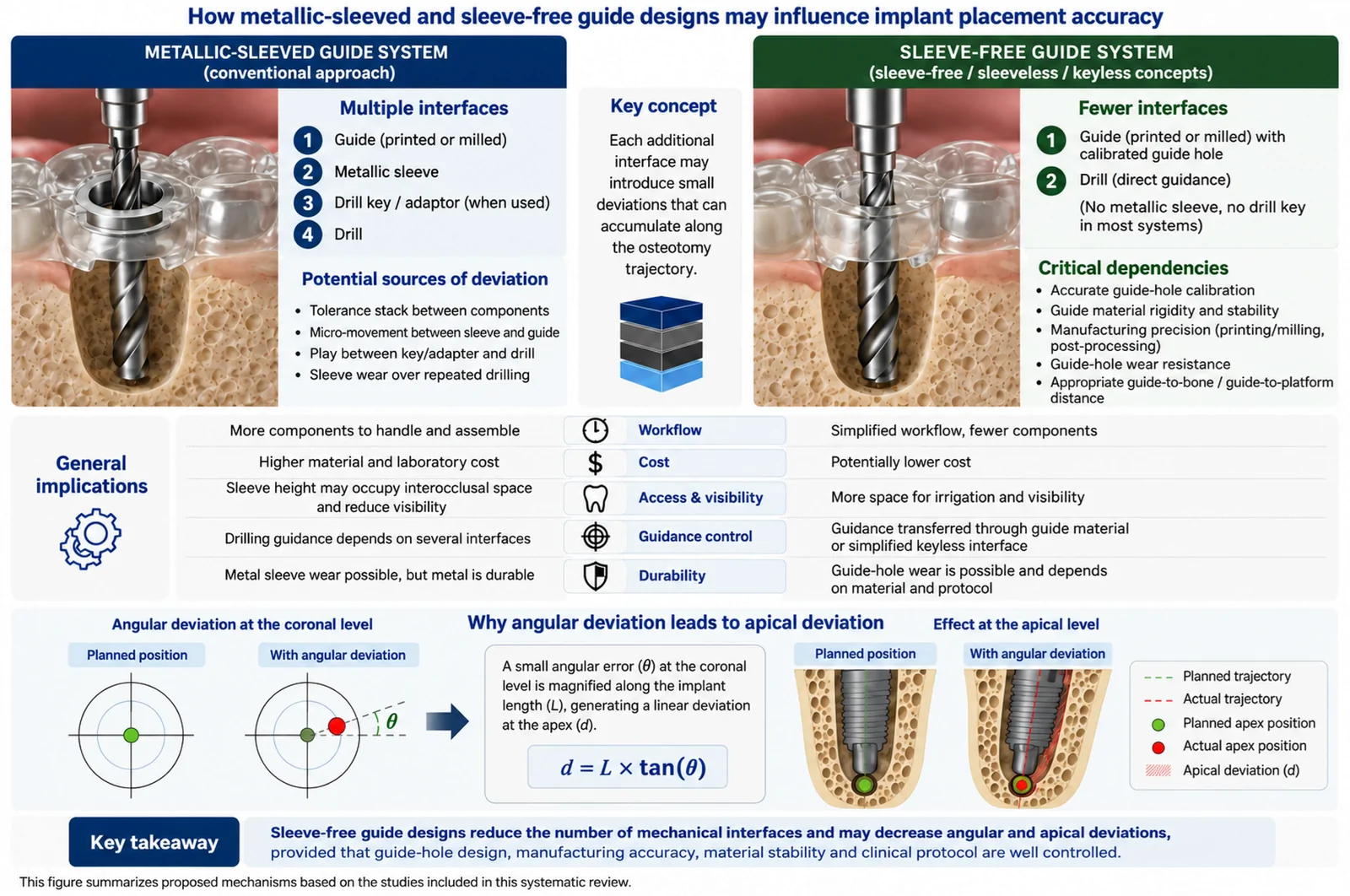
Proposed mechanical interpretation of metallic-sleeved and sleeve-free guide performance. The diagram contrasts guiding interfaces and workflow characteristics and illustrates how guide-to-platform distance and angular deviation may influence apical displacement.

Together, the sensitivity and leave-one-out analyses suggest that the direction of the main message is reasonably stable: sleeve-free guides appear to provide lower angular and apical deviations on average, while no statistically significant difference was detected for coronal/platform deviation. However, the high heterogeneity means that this conclusion should be interpreted as system- and context-dependent rather than universally applicable. The proposed mechanical interpretation of these findings is summarised in Figure 9.

### 4.7. Comparison with Previous Reviews

The findings are consistent with earlier evidence reporting similar or favourable accuracy for sleeve-free designs. Tallarico et al. previously reviewed templates with and without metallic sleeves in partial-arch restorations and reported favourable accuracy without sleeves while recognising methodological heterogeneity [28].

Esperouz et al. subsequently found no significant differences in angular or horizontal deviation between sleeved and sleeve-free guides [29]. The present review extends that synthesis through a broader and more recent evidence base, including 2025 and 2026 studies; separate analyses of angular, coronal/platform, and apical outcomes in their original clinical units; evaluation of healed and extraction-socket conditions; leave-one-out assessment of influential comparisons; and substitution analysis addressing the potential Raabe–Dulla overlap. It also integrates the identified methodological priorities into the SLEEVE Rule. These additions explain why the present review detected outcome-specific effects and greater heterogeneity while remaining compatible with the earlier conclusion that sleeve-free guides are a valid alternative.

The principal advance is therefore a more precise outcome-specific interpretation: no statistically significant pooled difference was detected for coronal/platform deviation, while sleeve-free systems showed small average angular and apical advantages.

### 4.8. Clinical Implications

Clinical relevance concerns both positional accuracy and workflow. Metallic sleeves may increase cost and laboratory steps, occupy interocclusal space, reduce visibility or irrigation, and add interfaces between the guide, sleeve, drill key, and drill [13,14,15,24]. These disadvantages are particularly relevant posteriorly, with limited mouth opening, or when direct access and irrigation are priorities.

Sleeve-free guides may simplify manufacturing and instrumentation, reduce mechanical interfaces, improve access, and permit more customized designs. The evidence indicates that, when properly designed and manufactured, they can achieve accurate implant placement and may improve angular and apical control in selected systems or conditions.

The pooled outcomes quantify technical implant-placement accuracy rather than clinical effectiveness. Their clinical relevance should therefore be interpreted according to the anatomical and prosthetic context while recognising that patient-centred outcomes were not assessed and no prespecified minimal clinically important difference was established for the evaluated deviation measures.

The pooled differences of −0.61° and −0.27 mm are modest in magnitude, and their practical relevance is likely to depend on case-specific anatomical and prosthetic margins. They may become more relevant in anatomically or prosthetically demanding situations, including narrow ridges, proximity to critical structures, immediate extraction sockets, limited interocclusal space, or critical screw-access trajectories. The findings should therefore be interpreted as a potential advantage in selected indications rather than universal clinical superiority.

Clinical decisions should consider the complete guided system, including support, guide-hole calibration, manufacturing method, material properties, implant site, sleeve-to-bone distance, and operator experience. The key message is not that metallic sleeves should be abandoned, but that their routine necessity should be reconsidered within modern digital workflows.

Sleeve-free guides may be particularly attractive when access, visibility, irrigation, or interocclusal space is limited and when a stable guide and validated guide-hole calibration are available. Metallic-sleeved systems may remain useful when a wear-resistant interface or system-specific validation is preferred.

### 4.9. Limitations

This review has several limitations. First, most included studies were in vitro. Although in vitro studies allow high experimental control and direct comparison of guide designs under standardised conditions, they cannot fully reproduce clinical variables such as saliva, bleeding, soft-tissue interference, patient movement, restricted access, surgical fatigue, and intraoperative guide instability. Nevertheless, the predominance of in vitro evidence is partly inherent to this research question. In vivo studies are more difficult to design because the same implant site cannot be treated twice under identical anatomical, surgical, and prosthetic conditions using both a metallic-sleeved and a sleeve-free guide. Moreover, patient-related factors such as bone density, ridge morphology, mouth opening, guide support, operator access, and surgical visibility may confound the isolated effect of guide design. Therefore, controlled in vitro models remain important for evaluating the mechanical contribution of the metallic sleeve, guide-hole geometry, drilling system, and manufacturing protocol. However, because clinical validation remains limited, the available evidence provides little information on long-term clinical outcomes, surgical complications, patient-related factors, and the durability of sleeve-free guide performance under routine clinical conditions.

Second, substantial heterogeneity was present across all outcomes. Differences in guide design, implant system, manufacturing technology, guide support, sleeve-to-bone distance, implant site morphology, planning and measurement software, measurement method, and outcome definition likely contributed to this heterogeneity. Consequently, the pooled estimates should be interpreted as average effects across diverse systems rather than as universal values applicable to all guided surgery protocols. Although several potential effect modifiers were considered qualitatively, including healed versus extraction sites, clinical versus in vitro setting, guide support, sleeve-to-bone distance, manufacturing method, and guide design, they could not be incorporated into formal subgroup analyses or meta-regression because they were inconsistently reported, unevenly distributed across studies, or represented by too few comparisons within each outcome.

Third, some relevant studies could not be included in the primary meta-analysis. The study by Ballesteros et al. was included only in sensitivity analyses because standard deviations had to be estimated from interquartile ranges [26]. The study by Adams et al. was retained in the qualitative synthesis because it reported mainly directional and signed deviations rather than directly comparable global angular, coronal/platform, and apical deviation outcomes [25]. Oh et al. and Aboul Azayem et al. reported more limited outcome sets, which restricted their contribution to quantitative synthesis [16,24].

Fourth, possible methodological overlap between Raabe et al. and Dulla et al. required conservative handling in the meta-analysis [17,18]. These studies were therefore used alternatively rather than simultaneously to avoid double counting related experimental conditions. Finally, publication bias was not formally assessed because fewer than 10 comparisons were available for each primary outcome, making funnel plot interpretation and statistical asymmetry tests unreliable.

Study-specific ICCs and sufficiently detailed cluster-adjusted variance estimates were not available from the published primary reports; therefore, the influence of within-unit dependence was evaluated through ICC-based sensitivity analyses rather than an exact cluster-adjusted reconstruction.

### 4.10. Toward Standardised Future Research: The SLEEVE Rule

The present review identified recurring inconsistencies in terminology, guide-system definition, anatomical and experimental conditions, manufacturing workflows, outcome measurement, and statistical reporting. Future comparative investigations should complement the core metallic-sleeved versus sleeve-free comparison with detailed reporting of the complete guided system. Particular attention should be given to guide support and fixation, seating verification, healed-ridge versus extraction-socket conditions, open versus closed guide-hole geometry, guide-to-platform or guide-to-bone distance, drill-key and instrumentation interfaces, manufacturing material and method, post-processing, calibration, guide-hole wear, and the system-specific drilling and implant-placement protocol.

The SLEEVE Rule was developed from recurring methodological and reporting patterns identified across the evidence evaluated in this review and is proposed as a structured framework for future comparative research. Its purpose is to provide a structured basis for standardising key methodological domains and improving the design, reporting, and comparability of future studies. The acronym comprises six methodological domains: Support and stabilisation; Local anatomy and site conditions; Exact guide-system definition; Endpoint definition and measurement; Validated workflow; and Evidence quality and analysis. Together, these domains provide a structured basis for planning future studies, interpreting system-dependent findings, and distinguishing effects associated with sleeve configuration from those arising from anatomical conditions, guide support, manufacturing, instrumentation, outcome measurement, or statistical analysis.

The framework also establishes a recommended minimum reporting set comprising guide-system definition, support type, site morphology, measurement method, manufacturing protocol, and the unit of analysis. Studies should distinguish the number of independent experimental units from the number of implant/site observations and report how within-unit clustering was handled when multiple observations arise from the same experimental unit. Angular, coronal/platform, and apical deviations should be reported separately in their original units, together with the group-level sample size, mean, and standard deviation.

**Figure 10:**
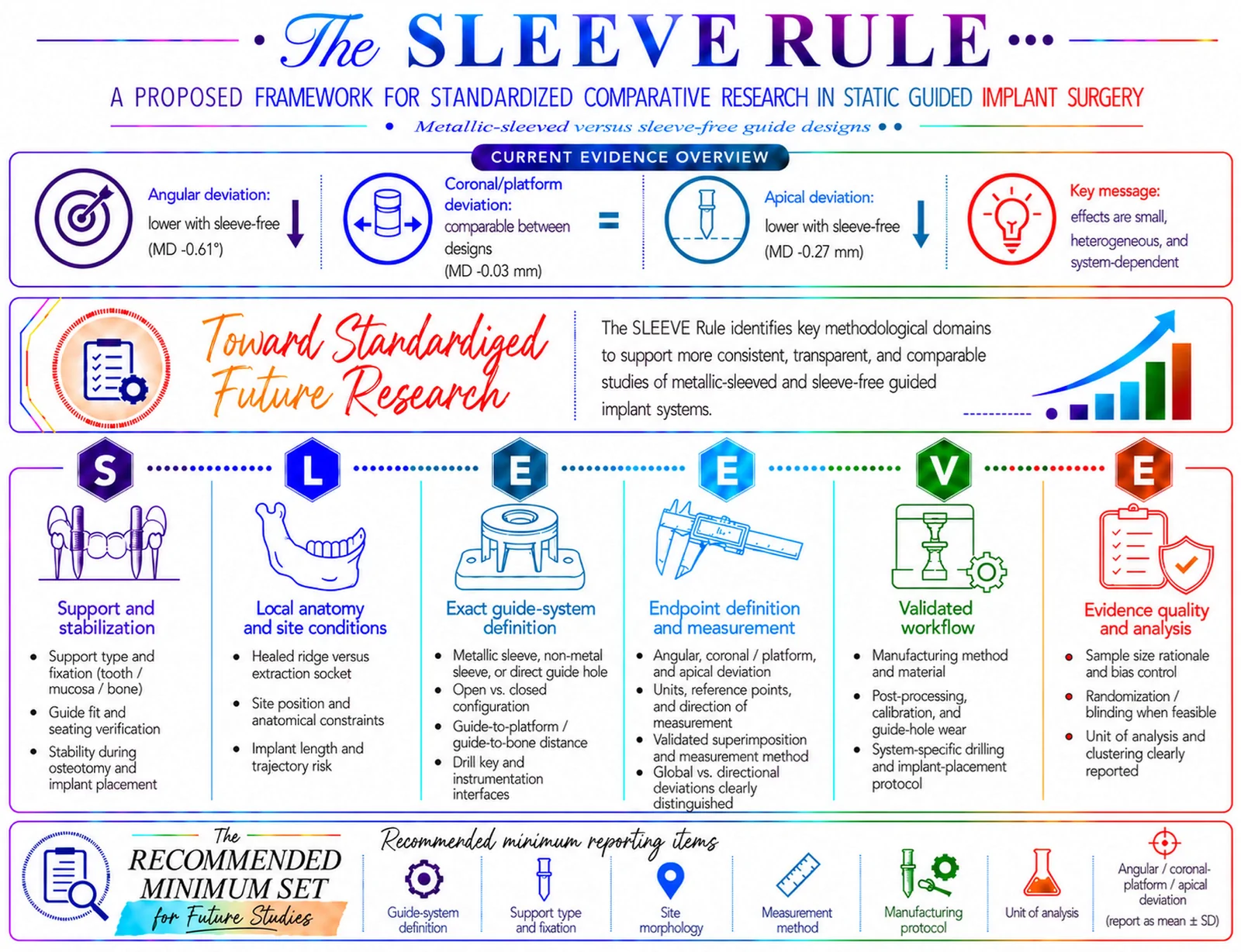
The SLEEVE Rule: a proposed framework for standardised comparative research in static guided implant surgery. The figure integrates the pooled findings with six methodological domains: Support and stabilisation; Local anatomy and site conditions; Exact guide-system definition; Endpoint definition and measurement; Validated workflow; and Evidence quality and analysis. Angular and apical pooled estimates favoured sleeve-free guides, whereas no statistically significant pooled difference was detected for coronal/platform deviation. MD, mean difference; SD, standard deviation.

Consistent reporting of these variables would improve reproducibility, facilitate direct comparison between studies, and strengthen future subgroup analyses and meta-analyses. Prospective clinical investigations should additionally examine whether the observed technical differences affect surgical access, irrigation, guide wear, anatomical safety, prosthetic outcomes, peri-implant conditions, or complications. The current pooled evidence and the complete SLEEVE Rule are summarised in Figure 10.

## 5. Conclusions

Within the limitations of this systematic review and meta-analysis, sleeve-free designs showed modest average reductions in angular and apical deviation, while no statistically significant difference was detected for coronal/platform deviation. Because heterogeneity was substantial to high and prediction intervals were broad, the pooled effects should be interpreted in relation to the characteristics of the complete guided system and study setting.

Metallic sleeves should therefore not be regarded as automatically mandatory in all static computer-assisted implant surgery workflows. When guide design, guide-hole calibration, manufacturing accuracy, material stability, support, and surgical protocol are properly controlled, sleeve-free guides may provide reliable guidance while reducing mechanical interfaces and facilitating access, visibility, irrigation, or instrumentation in selected situations.

The observed angular and apical advantages support the mechanical rationale that fewer guide–sleeve–key–drill interfaces may improve trajectory control in some systems. Further well-designed clinical studies are required to determine the practical relevance of these technical accuracy differences under routine clinical conditions. The SLEEVE Rule provides a structured framework for improving methodological consistency, transparent reporting, and comparability in future research on metallic-sleeved and sleeve-free guided implant systems.

## Figures and Tables

**Table 1:** PICOS framework of the systematic review.

PICOS Element	Definition
Population	Clinical participants or experimental models with dental implant sites undergoing static computer-assisted implant placement.
Intervention	Static computer-assisted implant placement performed without a metallic guiding sleeve.
Comparison	Static computer-assisted implant placement performed with a metallic guiding sleeve.
Outcomes	Implant-placement accuracy assessed through angular, coronal/platform, and apical deviation; three-dimensional/global deviation and other accuracy parameters were additionally recorded when reported.
Study design	Comparative in vitro, ex vivo, preclinical, or clinical studies evaluating static guided dental implant placement.

**Table 2:** Search strategy used for each database.

Database	Search Equation	Date Range
PubMed/MEDLINE	(dental implant* OR implant placement) AND (surgical guide* OR surgical template* OR static guidance OR computer-assisted implant surgery OR computer-aided implant surgery OR sCAIS OR template*) AND (sleeve OR sleeved OR sleeve-free OR sleeveless OR metal sleeve OR metallic sleeve OR open sleeve OR closed sleeve) AND (accuracy OR trueness OR precision OR deviation OR angular)	2016–2026
Scopus	((“dental implant*” OR “implant placement”) AND (“surgical guide*” OR “surgical template*” OR “guided implant surgery” OR “computer-assisted implant surgery” OR “computer-aided implant surgery” OR “static computer-assisted implant surgery” OR sCAIS) AND (sleeve OR sleeved OR “sleeve-free” OR sleeveless OR “metal sleeve” OR “metallic sleeve” OR “open sleeve” OR “closed sleeve”) AND (accuracy OR trueness OR precision OR deviation OR angular))	2016–2026
Web of Science	(dental implant* OR implant placement) AND (surgical guide* OR surgical template* OR static guidance OR computer-assisted implant surgery OR computer-aided implant surgery OR sCAIS OR template*) AND (sleeve OR sleeved OR sleeve-free OR sleeveless OR metal sleeve OR metallic sleeve OR open sleeve OR closed sleeve) AND (accuracy OR trueness OR precision OR deviation OR angular)	2016–2026
Cochrane Library/CENTRAL	(dental implant* OR implant placement) AND (surgical guide* OR surgical template* OR static guidance OR computer-assisted implant surgery OR computer-aided implant surgery OR sCAIS OR template*) AND (sleeve OR sleeved OR sleeve-free OR sleeveless OR metal sleeve OR metallic sleeve OR open sleeve OR closed sleeve) AND (accuracy OR trueness OR precision OR deviation OR angular)	2016–2026
Google Scholar	“static computer-assisted implant surgery” sleeveless accuracy	2016–2026

**Table 3:** Eligibility criteria.

Inclusion Criteria	Exclusion Criteria
In vitro, ex vivo, preclinical, or clinical studies evaluating the accuracy of surgical guides for dental implant placement.	Narrative reviews, editorials, letters to the editor, comments, errata without original data, or previous systematic reviews used as primary studies.
Studies based on static guided implant surgery.	Studies without direct comparison between metallic-sleeved and sleeveless/sleeve-free surgical guides.
Studies directly comparing metallic-sleeved surgical guides with surgical guides without metallic sleeves or equivalent sleeve-free/sleeveless designs.	Studies focused exclusively on dynamic navigation.
Studies reporting at least one quantitative implant placement accuracy outcome, including angular deviation, coronal/platform deviation, apical deviation, or three-dimensional/global deviation.	Studies evaluating non-implant surgical guides.
Studies reporting numerical implant-placement accuracy data for direct comparative assessment.	Studies without extractable numerical implant-placement accuracy data for the comparison of interest.
	Studies in which the isolated effect of the presence or absence of a metallic sleeve could not be determined.
	Studies comparing only open-sleeve versus closed-sleeve designs when both groups used a sleeve and no true sleeve-free group was present.

**Table 4:** Data extraction variables.

Category	Data Extracted
Identification	Internal ID, author, year, title.
Study design	In vitro, ex vivo, clinical, prospective, retrospective, randomized controlled trial.
Sample	Type of sample, model, or population; number of implants, implant sites, or patients.
Guide characteristics	Type of guide support, metallic-sleeved group, sleeve-free/sleeveless group, guide design.
Comparison	Presence of direct comparison between guides with and without metallic sleeves.
Measurement method	CBCT, STL, scan body, laboratory scanner, superimposition software, or equivalent method.
Quantitative outcomes	Mean, standard deviation, and sample size for angular, coronal/platform, apical, and three-dimensional/global deviation when available.
Units	Degrees for angular deviation; millimeters for linear deviations.
Study conclusion	Main conclusion reported by the authors.
Inclusion decision	Qualitative synthesis, main meta-analysis, sensitivity analysis, or exclusion from meta-analysis.
Methodological notes	Data comparability, missing data, conversion, or possible overlap.

**Table 5:** Characteristics of the included studies and comparative guided-surgery protocols.

Study	Design/Setting	Sample/Study Unit	Site Condition	Comparative Guide Concepts	Measurement Method	Accuracy Outcomes Reported
Chung et al. [15]	In vitro	49 partially edentulous mandibular models; 98 implant placements	Standardised implant sites	Metallic-sleeved vs. non-metal/sleeve-free template designs	STL-based 3D comparison	Coronal, apical, vertical, and angular deviation
Sittikornpaiboon et al. [11]	In vitro simulation-based study	25 3D-printed models; 50 implants	Standardised edentulous sites	Integrated sleeve-on-drill guidance with vs. without metallic sleeve	CBCT/3D digital comparison	Platform, apical, and angular deviation
Dulla et al. [17]	In vitro	18 partially edentulous maxillary models; 72 implants	Fully healed ridges and fresh extraction sockets	Manufacturer’s sleeve vs. sleeveless guide-hole design	3D digital superimposition	Crestal, apical, and angular deviation
Galli et al. [14]	In vitro	30 maxillary experimental casts; 210 implant sites	Healed sites and fresh extraction sockets	Metallic-sleeved vs. sleeve-free static guidance	3D digital superimposition	Coronal, apical, depth, and angular deviation
Raabe et al. [18]	In vitro	18 partially edentulous maxillary models; 108 implants	Healed alveolar ridges and extraction sockets	Manufacturer’s sleeve vs. sleeveless guide-hole design	3D digital deviation assessment	Crestal, apical, and angular deviation
Oh et al. [24]	In vitro	20 mandibular typodonts; 60 implant placements	Standardised implant sites	Metal sleeve-incorporated vs. metal sleeve-free 3D-printed guides	Digital angular assessment and guide material assessment	Directional angular deviation and guide-related outcomes
Ballesteros et al. [26]	In vitro	12 models; 72 implants	Standardised implant sites	Metallic-sleeved vs. sleeve-free guides produced by different manufacturing methods	Digital 3D comparison	3D crestal, 3D apical, and angular deviation
Adams et al. [25]	In vitro	20 casts per experimental step; 10 per group	Standardised single edentulous site	Guides with vs. without metallic sleeves	Postoperative CBCT superimposition	Directional cervical, apical, mesial, and angular deviation
Hang and Guentsch [12]	In vitro	120 mandibular replicas; 60 per group	Standardised implant site; 2-, 4-, and 6-mm guide-to-platform distances	Metallic-sleeved vs. sleeve-free guidance	Scan-body/laboratory-scan comparison	Platform, apical, and angular deviation
Tallarico et al. [13]	Randomized controlled trial	30 patients; 90 implants	Partially edentulous clinical sites	Static templates with vs. without metallic sleeves	Planned/postoperative digital comparison	Angular, horizontal, and vertical deviation
Aboul Azayem et al. [16]	In vitro	10 mandibular Kennedy Class III models; 20 implants	Premolar and molar sites	Metal sleeve-incorporated vs. metal sleeve-free guide	Digital superimposition	Angular deviation

**Table 6:** Outcome-specific quantitative data and analytical inclusion of the included studies.

Study/Comparison	Angular Deviation	Coronal/Platform Deviation	Apical Deviation	Role in Synthesis
Chung et al. [15]	S: 1.04 ± 0.55° (*n* = 14); SF: 1.05 ± 0.56° (*n* = 14)	S: 0.36 ± 0.12 mm; SF: 0.36 ± 0.12 mm	S: 0.39 ± 0.09 mm; SF: 0.40 ± 0.09 mm	Primary meta-analysis: angular, coronal/platform, and apical deviation.
Sittikornpaiboon et al. [11]	S: 5.13 ± 1.86° (*n* = 10); SF: 5.30 ± 1.04° (*n* = 10)	S: 1.09 ± 0.12 mm; SF: 0.81 ± 0.15 mm	S: 1.95 ± 0.48 mm; SF: 1.73 ± 0.23 mm	Primary meta-analysis: angular, coronal/platform, and apical deviation.
Dulla et al. [17]	S: 3.46 ± 1.90° (*n* = 36); SF: 2.72 ± 1.93° (*n* = 36)	S: 0.63 ± 0.30 mm; SF: 0.54 ± 0.34 mm	S: 1.27 ± 0.67 mm; SF: 1.02 ± 0.66 mm	Sensitivity analysis: substituted for Raabe et al. for angular, coronal/platform, and apical deviation.
Galli et al. [14], extraction sockets	S: 4.63 ± 2.75° (*n* = 30); SF: 3.80 ± 1.67° (*n* = 30)	S: 0.69 ± 0.40 mm; SF: 1.08 ± 0.54 mm	S: 1.86 ± 0.98 mm; SF: 1.54 ± 0.68 mm	Primary meta-analysis; healed and extraction conditions entered separately. Study-level sensitivity analysis combined both conditions.
Galli et al. [14], healed sites	S: 4.42 ± 1.51° (*n* = 40); SF: 2.31 ± 1.30° (*n* = 40)	S: 0.73 ± 0.20 mm; SF: 0.62 ± 0.30 mm	S: 1.74 ± 0.48 mm; SF: 0.98 ± 0.53 mm	Primary meta-analysis; healed and extraction conditions entered separately. Study-level sensitivity analysis combined both conditions.
Raabe et al. [18]	S: 3.30 ± 1.90° (*n* = 36); SF: 2.60 ± 1.60° (*n* = 36)	S: 0.60 ± 0.30 mm; SF: 0.50 ± 0.30 mm	S: 1.20 ± 0.70 mm; SF: 1.00 ± 0.50 mm	Primary meta-analysis; replaced by Dulla et al. in the substitution sensitivity analysis.
Oh et al. [24]	Site- and direction-specific angular outcomes; no single global value used for pooling.	Not reported as a directly comparable global outcome.	Not reported as a directly comparable global outcome.	Qualitative synthesis only; outcome format not directly comparable with the pooled global measures.
Ballesteros et al. [26]	S: 3.883° (IQR 2.850); SF: 1.442° (IQR 1.567)	S: 1.028 mm (IQR 0.424); SF: 0.660 mm (IQR 0.572)	S: 1.360 mm (IQR 0.990); SF: 0.840 mm (IQR 0.620)	Sensitivity analysis: angular, coronal/platform, and apical deviation after IQR-to-SD approximation.
Adams et al. [25]	Directional/signed angular outcomes reported; not pooled as a global measure.	Directional/signed cervical and mesial outcomes reported; not pooled as a global measure.	Directional/signed apical outcomes reported; not pooled as a global measure.	Qualitative synthesis only; predominantly directional/signed outcomes were not directly comparable with the pooled global outcomes.
Hang and Guentsch [12]	S: 2.41 ± 1.41° (*n* = 60); SF: 1.65 ± 0.93° (*n* = 60)	S: 0.36 ± 0.17 mm; SF: 0.30 ± 0.15 mm	S: 0.74 ± 0.34 mm; SF: 0.53 ± 0.31 mm	Primary meta-analysis: angular, coronal/platform, and apical deviation.
Tallarico et al. [13]	S: 2.25 ± 1.41° (*n* = 41); SF: 1.98 ± 2.38° (*n* = 49)	S: 0.52 ± 0.30 mm; SF: 0.61 ± 0.49 mm	Not reported separately.	Primary meta-analysis: angular and coronal/platform deviation; excluded in the in-vitro-only study-setting sensitivity analysis.
Aboul Azayem et al. [16]	S: 2.24 ± 0.36° (*n* = 10); SF: 1.98 ± 0.23° (*n* = 10)	Not reported.	Not reported.	Primary meta-analysis: angular deviation.

Note. S, metallic-sleeved; SF, sleeve-free. Values are mean ± SD unless otherwise indicated. Ballesteros et al. [26] reported medians and interquartile ranges (IQR).

**Table 7:** Primary random-effects meta-analysis results.

Outcome	No. of Comparisons	Pooled Mean Difference	95% CI	*p*-Value	Heterogeneity	95% Prediction Interval	Direction of Effect	Interpretation
Angular deviation, °	8	−0.61°	−1.12 to −0.10	0.019	I^2^ = 81.7% τ^2^ = 0.405 Q [7] = 38.35; *p* < 0.001	−1.96 to 0.73°	Favours sleeve-free	Lower angular deviation on average; substantial heterogeneity.
Coronal/platform deviation, mm	7	−0.03 mm	−0.16 to 0.11	0.689	I^2^ = 81.6% τ^2^ = 0.028 Q [6] = 32.58; *p* < 0.001	−0.38 to 0.33 mm	No clear direction	No statistically significant difference was found between sleeve-free and sleeved guides.
Apical deviation, mm	6	−0.27 mm	−0.50 to −0.05	0.017	I^2^ = 90.0% τ^2^ = 0.063 Q [5] = 49.98; *p* < 0.001	−0.82 to 0.27 mm	Favours sleeve-free	Lower apical deviation on average; high heterogeneity.

Note. Random-effects models were used for all outcomes. Mean differences were calculated as sleeve-free minus sleeved; therefore, negative values indicate lower deviation in the sleeve-free group. CI, confidence interval; I^2^, inconsistency statistic.

**Table 8:** Sensitivity analyses.

Outcome	Analysis	No. of Comparisons	Pooled Mean Difference	95% CI	*p*-Value	I^2^	Interpretation
Angular deviation, °	Primary analysis	8	−0.61°	−1.12 to −0.10	0.019	81.7%	Significant average effect; substantial heterogeneity.
Angular deviation, °	Ballesteros added	9	−0.79°	−1.37 to −0.22	0.007	85.2%	Significant; heterogeneity remained high.
Angular deviation, °	Dulla replaced Raabe	8	−0.62°	−1.13 to −0.10	0.019	81.8%	Essentially unchanged; substantial heterogeneity persisted.
Angular deviation, °	Tallarico et al. [13] excluded	7	−0.66°	−1.23 to −0.08	0.025	84.2%	Significant; direction and interpretation unchanged.
Angular deviation, °	Galli site conditions combined	7	−0.51°	−0.93 to −0.09	0.018	72.9%	Significant; direction and interpretation unchanged.
Coronal/platform deviation, mm	Primary analysis	7	−0.03 mm	−0.16 to 0.11	0.689	81.6%	No significant difference.
Coronal/platform deviation, mm	Ballesteros added	8	−0.06 mm	−0.20 to 0.08	0.388	82.7%	No significant difference; direction unchanged.
Coronal/platform deviation, mm	Dulla replaced Raabe	7	−0.03 mm	−0.16 to 0.11	0.706	81.5%	Unchanged.
Coronal/platform deviation, mm	Tallarico et al. [13] excluded	6	−0.04 mm	−0.20 to 0.11	0.574	82.9%	No significant difference; interpretation unchanged.
Coronal/platform deviation, mm	Galli site conditions combined	6	−0.04 mm	−0.15 to 0.07	0.426	79.3%	No significant difference; interpretation unchanged.
Apical deviation, mm	Primary analysis	6	−0.27 mm	−0.50 to −0.05	0.017	90.0%	Significant average effect; high heterogeneity.
Apical deviation, mm	Ballesteros added	7	−0.30 mm	−0.51 to −0.10	0.004	89.2%	Significant; heterogeneity remained high.
Apical deviation, mm	Dulla replaced Raabe	6	−0.28 mm	−0.51 to −0.06	0.015	90.1%	Essentially unchanged; high heterogeneity persisted.
Apical deviation, mm	Galli site conditions combined	5	−0.22 mm	−0.42 to −0.03	0.025	86.9%	Significant; direction and interpretation unchanged.

Note. Mean differences were calculated as sleeve-free minus sleeved. Negative values indicate lower deviation in the sleeve-free group. Ballesteros et al. [26] was included only in sensitivity analyses because standard deviations were estimated from interquartile ranges. Dulla et al. [17] was used as a replacement for Raabe et al. [18] because both studies may share methodological or experimental overlap.

**Table 9:** Summary of leave-one-out analysis.

Outcome	Leave-One-Out Finding	Most Influential Omission	Effect on Pooled Estimate	Effect on Heterogeneity	Interpretation
Angular deviation	Direction remained in favour of sleeve-free guides.	Galli et al. [14], healed sites	MD changed from −0.61° to −0.36°	I^2^ decreased from 81.7% to 32.4%	Influential subgroup; retained because eligible and clinically relevant.
Coronal/platform deviation	Conclusion remained non-significant.	Galli et al. [14], extraction sockets	MD changed from −0.03 mm to −0.08 mm	I^2^ decreased from 81.6% to 73.3%	No statistically significant difference; pooled effect remained close to zero.
Apical deviation	Direction remained in favour of sleeve-free guides.	Galli et al. [14], healed sites	MD changed from −0.27 mm to −0.14 mm	I^2^ decreased from 90.0% to 71.2%	Magnitude influenced by Galli; direction remained favourable.

Note. Leave-one-out analysis was performed by repeating each meta-analysis after removing one comparison at a time. Mean differences were calculated as sleeve-free minus sleeved.

## Data Availability

All data generated or analysed during this systematic review and meta-analysis are included in this article and its Supplementary Materials Files. The review protocol was registered in the Open Science Framework.

## Supplementary Materials

The following supporting information can be downloaded at: https://www.mdpi.com/article/10.3390/jcm15207935/s1, Table S1: PRISMA 2020 checklist [30]; Table S2: GRADE assessment of the certainty of evidence [34,35]; Table S3: Full-text reports excluded after eligibility assessment and reasons for exclusion [10,20,21,22,28,29]; Table S4: Statistical unit and availability of information relevant to within-cluster dependence in studies contributing to quantitative synthesis [11,12,13,14,15,16,17,18,26,37]; Table S5: Sensitivity of pooled estimates to assumed within-cluster correlation [14,37].

## References

[1] Schneider D., Marquardt P., Zwahlen M., Jung R.E. (2009). A systematic review on the accuracy and clinical outcome of computer-guided template-based implant dentistry. Clin. Oral Implant. Res..

[2] Jung R.E., Schneider D., Ganeles J., Wismeijer D., Zwahlen M., Hammerle C.H.F., Tahmaseb A. (2009). Computer technology applications in surgical implant dentistry: A systematic review. Int. J. Oral Maxillofac. Implant..

[3] D’haese J., Ackhurst J., Wismeijer D., De Bruyn H., Tahmaseb A. (2017). Current state of the art of computer-guided implant surgery. Periodontol. 2000.

[4] Tahmaseb A., Wu V., Wismeijer D., Coucke W., Evans C. (2018). The accuracy of static computer-aided implant surgery: A systematic review and meta-analysis. Clin. Oral Implant. Res..

[5] Van Assche N., Vercruyssen M., Coucke W., Teughels W., Jacobs R., Quirynen M. (2012). Accuracy of computer-aided implant placement. Clin. Oral Implant. Res..

[6] Bover-Ramos F., Viña-Almunia J., Cervera-Ballester J., Peñarrocha-Diago M., García-Mira B. (2018). Accuracy of implant placement with computer-guided surgery: A systematic review and meta-analysis comparing cadaver, clinical, and in vitro studies. Int. J. Oral Maxillofac. Implant..

[7] Zhou W., Liu Z., Song L., Kuo C.-L., Shafer D.M. (2018). Clinical factors affecting the accuracy of guided implant surgery: A systematic review and meta-analysis. J. Evid. Based Dent. Pract..

[8] Putra R.H., Yoda N., Astuti E.R., Sasaki K. (2022). The accuracy of implant placement with computer-guided surgery in partially edentulous patients and possible influencing factors: A systematic review and meta-analysis. J. Prosthodont. Res..

[9] Vercruyssen M., Coucke W., Naert I., Jacobs R., Teughels W., Quirynen M. (2015). Depth and lateral deviations in guided implant surgery: An RCT comparing guided surgery with mental navigation or the use of a pilot-drill template. Clin. Oral Implant. Res..

[10] Gargallo-Albiol J., Barootchi S., Salomó-Coll O., Wang H.-L. (2019). Advantages and disadvantages of implant navigation surgery: A systematic review. Ann. Anat..

[11] Sittikornpaiboon P., Arunjaroensuk S., Kaboosaya B., Subbalekha K., Mattheos N., Pimkhaokham A. (2021). Comparison of the accuracy of implant placement using different drilling systems for static computer-assisted implant surgery: A simulation-based experimental study. Clin. Implant Dent. Relat. Res..

[12] Hang J., Guentsch A. (2025). Are sleeves necessary in static computer-assisted implant surgery? A comparative in vitro analysis. Clin. Oral Implant. Res..

[13] Tallarico M., Martinolli M., Kim Y.J., Cocchi F., Meloni S.M., Alushi A., Xhanari E. (2019). Accuracy of computer-assisted template-based implant placement using two different surgical templates designed with or without metallic sleeves: A randomized controlled trial. Dent. J..

[14] Galli M., Mendonça G., Meneghetti P., Bekkali M., Travan S., Wang H.L., Li J. (2023). Sleeveless guided implant placement compared to conventional approaches: An in vitro study at healed sites and fresh extraction sockets. Int. J. Oral Implantol..

[15] Chung J., Park J., Son H., Hong S.J., Pae A. (2025). Influence of template design on the accuracy of static computer-assisted implant surgery. J. Adv. Prosthodont..

[16] Aboul Azayem Y.M., Helmy M.A., Hussein S.K. (2026). Evaluation of angular deviation in metal sleeve-free vs metal sleeve-incorporated 3D-printed computer-assisted implant surgical guides in mandibular Kennedy class III: In vitro study. Ain Shams Dent. J..

[17] Dulla F.A., Couso-Queiruga E., Chappuis V., Yilmaz B., Abou-Ayash S., Raabe C. (2023). Influence of alveolar ridge morphology and guide-hole design on the accuracy of static computer-assisted implant surgery with two implant macro-designs: An in vitro study. J. Dent..

[18] Raabe C., Schuetz T.S., Chappuis V., Yilmaz B., Abou-Ayash S., Couso-Queiruga E. (2023). Accuracy of keyless vs drill-key implant systems for static computer-assisted implant surgery using two guide-hole designs compared to freehand implant placement: An in vitro study. Int. J. Implant Dent..

[19] Alevizakos V., Stryga Y., von See C. (2024). Enhancing implant position accuracy in guided implantology: The role of drill play under lateral forces. Clin. Exp. Dent. Res..

[20] Chen Z., Li J., Wei C.X., Mendonça G., Wang H.L. (2024). Accuracy of open-sleeved vs closed-sleeved static computer-assisted implant systems in immediate maxillary molar implant placement: An in vitro study. Clin. Oral Implant. Res..

[21] Li J., Meneghetti P.C., Galli M., Mendonça G., Chen Z., Wang H.L. (2022). Open-sleeve templates for computer-assisted implant surgery at healed or extraction sockets: An in vitro comparison to closed-sleeve guided system and free-hand approach. Clin. Oral Implant. Res..

[22] Guentsch A., An H., Dentino A.R. (2022). Precision and trueness of computer-assisted implant placement using static surgical guides with open and closed sleeves: An in vitro analysis. Clin. Oral Implant. Res..

[23] Abdallah S.A., Kaddah A., Elkhadem A. (2021). The accuracy of implant placement using opened versus closed sleeve computer surgical guide: A split mouth technique. Ain Shams Dent. J..

[24] Oh K.C., Shim J.S., Park J.M. (2021). In vitro comparison between metal sleeve-free and metal sleeve-incorporated 3D-printed computer-assisted implant surgical guides. Materials.

[25] Adams C.R., Ammoun R., Deeb G.R., Bencharit S. (2023). Influence of metal guide sleeves on the accuracy and precision of dental implant placement using guided implant surgery: An in vitro study. J. Prosthodont..

[26] Ballesteros J., Vásquez S., Revilla-León M., Gómez-Polo M. (2025). A comparative study on the accuracy of implant placement using 3D-printed and milled guides without metal sleeves. Clin. Implant Dent. Relat. Res..

[27] Sharma S., Tan E., Tran B., Siow H.Y., Tafesse E., Thong Y.H.J., Tan R.J.M., Son J., Todaro L., Teo J. (2024). Effect of pilot-guided implant placement concept on the accuracy of osteotomy preparation and implant placement. J. Oral Sci..

[28] Tallarico M., Czajkowska M., Cicciù M., Giardina F. (2021). Accuracy of surgical templates with and without metallic sleeves in case of partial arch restorations: A systematic review. J. Dent..

[29] Esperouz F., Almas K., Alajmi M., Almutairi M., Alotaibi F. (2025). Accuracy of guided implant placement using surgical guides with or without metal sleeves: Systematic review and meta-analysis. Int. J. Oral Implantol..

[30] Page M.J., McKenzie J.E., Bossuyt P.M., Boutron I., Hoffmann T.C., Mulrow C.D., Shamseer L., Tetzlaff J.M., Akl E.A., Brennan S.E. (2021). The PRISMA 2020 statement: An updated guideline for reporting systematic reviews. BMJ.

[31] Page M.J., Moher D., Bossuyt P.M., Boutron I., Hoffmann T.C., Mulrow C.D., Shamseer L., Tetzlaff J.M., Akl E.A., Brennan S.E. (2021). PRISMA 2020 explanation and elaboration: Updated guidance and exemplars for reporting systematic reviews. BMJ.

[32] Elliott T., Hamilton A., Griseto N., Gallucci G.O. (2022). Additively Manufactured Surgical Implant Guides: A Review. J. Prosthodont..

[33] Clark J.M., Sanders S., Carter M., Honeyman D., Cleo G., Auld Y., Booth D., Condron P., Dalais C., Bateup S. (2020). Improving the translation of search strategies using the Polyglot Search Translator: A randomized controlled trial. J. Med. Libr. Assoc..

[34] Sheth V.H., Shah N.P., Jain R., Bhanushali N., Bhatnagar V. (2024). Development and validation of a risk-of-bias tool for assessing in vitro studies conducted in dentistry: The QUIN. J. Prosthet. Dent..

[35] Higgins J.P.T., Savović J., Page M.J., Elbers R.G., Sterne J.A.C. (2019). Revised Cochrane Risk-of-Bias Tool for Randomized Trials (RoB 2).

[36] Viechtbauer W. (2010). Conducting meta-analyses in R with the metafor package. J. Stat. Softw..

[37] Higgins J.P.T., Eldridge S., Li T., Higgins J.P.T., Thomas J., Chandler J., Cumpston M., Li T., Page M.J., Welch V.A. (2024). Chapter 23: Including Variants on Randomized Trials (Last Updated October 2019). Cochrane Handbook for Systematic Reviews of Interventions, Version 6.5.

