# Supplementary material for: Accuracy of Metallic-Sleeved Versus Sleeve-Free Surgical Guides in Static Computer-Assisted Implant Surgery: A Systematic Review and Meta-Analysis
Source: J Clin Med. 2026 Oct 14;15(20):7935. doi: 10.3390/jcm15207935 (PMC13705713; doi:10.3390/jcm15207935)
Supplement: Supplementary file 1 [file jcm-15-07935-s001.zip › jcm-4556956-supplementary.pdf]

Supplementary Table S1. PRISMA 2020 checklist for the systematic review and meta-analysis.

PRISMA 2020 Checklist

Section and Topic	Item #	Checklist item	Location where item is reported
**TITLE**			
Title	**1**	Identify the report as a systematic review.	Title page, p. 1. The title identifies the work as a systematic review and meta-analysis.
**ABSTRACT**			
Abstract	**2**	See the PRISMA 2020 for Abstracts checklist.	Structured Abstract, pp. 2-3. Includes Introduction, Objectives, Materials and Methods, Results, Discussion, Conclusions, and keywords.
**INTRODUCTION**			
Rationale	**3**	Describe the rationale for the review in the context of existing knowledge.	Introduction, pp. 4-7. Rationale is provided in relation to sCAIS accuracy, metallic sleeves, sleeve-free guide designs, mechanical tolerances, and previous reviews.
Objectives	**4**	Provide an explicit statement of the objective(s) or question(s) the review addresses.	End of Introduction, pp. 7-8. General objective, specific objectives, PICO question, and hypotheses are stated.
**METHODS**			
Eligibility criteria	**5**	Specify the inclusion and exclusion criteria for the review and how studies were grouped for the syntheses.	Methods 2.4, p. 10, and Table 3. Inclusion and exclusion criteria are specified; grouping for synthesis is described in Methods 2.9 and Table 6.
Information sources	**6**	Specify all databases, registers, websites, organisations, reference lists and other sources searched or consulted to identify studies. Specify the date when each source was last searched or consulted.	Methods 2.3, pp. 8-9, and Table 2. Databases and complementary sources are listed: PubMed/MEDLINE, Scopus, Web of Science, Cochrane/CENTRAL, Google Scholar, and manual reference screening.
Search strategy	**7**	Present the full search strategies for all databases, registers and websites, including any filters and limits used.	Methods 2.3, pp. 8-9, and Table 2. Search equations, date range, and number of records for each database/source are reported.
Selection process	**8**	Specify the methods used to decide whether a study met the inclusion criteria of the review, including how many reviewers screened each record and each report retrieved, whether they worked independently, and if applicable, details of automation tools used in the process.	Methods 2.5, pp. 10-11, and Results 3.1/Figure 2, pp. 14-15. Screening, full-text assessment, disagreement resolution, and inter-examiner agreement are described.
Data collection process	**9**	Specify the methods used to collect data from reports, including how many reviewers collected data from each report, whether they worked independently, any processes for obtaining or confirming data from study investigators, and if applicable, details of automation tools used in the process.	Methods 2.6, pp. 11-12, and Table 4. Standardized extraction spreadsheet and extracted variables are described.
Data items	**10a**	List and define all outcomes for which data were sought. Specify whether all results that were compatible with each outcome domain in each study were sought (e.g. for all measures, time points, analyses), and if not, the methods used to decide which results to collect.	Methods 2.7, p. 12. Primary and secondary outcomes are listed and defined.

Section and Topic	Item #	Checklist item	Location where item is reported
Data items	**10b**	List and define all other variables for which data were sought (e.g. participant and intervention characteristics, funding sources). Describe any assumptions made about any missing or unclear information.	Methods 2.6, pp. 11-12, and Table 4. Study design, sample, guide support, guide characteristics, comparison group, measurement method, and methodological notes are listed.
Study risk of bias assessment	**11**	Specify the methods used to assess risk of bias in the included studies, including details of the tool(s) used, how many reviewers assessed each study and whether they worked independently, and if applicable, details of automation tools used in the process.	Methods 2.8, p. 12; Results 3.3, pp. 18-20; Figures 3 and 4. QUIN was used for in vitro studies and RoB 2 for the randomized controlled trial.
Effect measures	**12**	Specify for each outcome the effect measure(s) (e.g. risk ratio, mean difference) used in the synthesis or presentation of results.	Methods 2.9-2.10, pp. 13-14. Mean difference was used as the effect measure; direction was defined as sleeve-free minus sleeved.
Synthesis methods	**13a**	Describe the processes used to decide which studies were eligible for each synthesis (e.g. tabulating the study intervention characteristics and comparing against the planned groups for each synthesis (item #5)).	Methods 2.9, p. 13, and Results 3.2/Table 6, pp. 16-18. Eligibility for each quantitative synthesis and sensitivity analysis is described.
Synthesis methods	**13b**	Describe any methods required to prepare the data for presentation or synthesis, such as handling of missing summary statistics, or data conversions.	Methods 2.9 and 2.11, pp. 13-14. Data extraction, handling of distinct subgroups, IQR-to-SD conversion for sensitivity analysis, and approach to possible overlap are described.
Synthesis methods	**13c**	Describe any methods used to tabulate or visually display results of individual studies and syntheses.	Methods 2.6-2.7, pp. 11-12, and Results tables/figures, pp. 16-28. Study characteristics, risk of bias, forest plots, and synthesis tables are presented.
Synthesis methods	**13d**	Describe any methods used to synthesize results and provide a rationale for the choice(s). If meta-analysis was performed, describe the model(s), method(s) to identify the presence and extent of statistical heterogeneity, and software package(s) used.	Methods 2.10, pp. 13-14. Random-effects meta-analysis, mean differences, 95% confidence intervals, p-values, I2, and Q test are described.
Synthesis methods	**13e**	Describe any methods used to explore possible causes of heterogeneity among study results (e.g. subgroup analysis, meta-regression).	Methods 2.10-2.11, pp. 13-14; Results 3.5-3.7, pp. 22-28; Discussion 4.5, pp. 31-32. Heterogeneity assessment and exploration are reported.
Synthesis methods	**13f**	Describe any sensitivity analyses conducted to assess robustness of the synthesized results.	Methods 2.11, p. 8; Supplementary Tables S4–S5. Sensitivity analyses include Ballesteros conversion, Dulla/Raabe substitution, leave-one-out, study-setting, Galli-combined, and clustering/design-effect analyses.
Reporting bias assessment	**14**	Describe any methods used to assess risk of bias due to missing results in a synthesis (arising from reporting biases).	Methods 2.12, p. 14. Publication/reporting bias assessment was not formally performed because each meta-analysis included fewer than 10 comparisons.
Certainty assessment	**15**	Describe any methods used to assess certainty (or confidence) in the body of evidence for an outcome.	Methods 2.8, p. 12, and Supporting Information, p. 39. GRADE assessment is reported as Supplementary Table S2.
**RESULTS**			
Study selection	**16a**	Describe the results of the search and selection process, from the number of records identified in the search to the number of studies included in the review, ideally using a flow diagram.	Results 3.1, pp. 14-16, and Figure 2, p. 15. Identification, deduplication, screening, full-text assessment, exclusions, and included studies are reported.
Study selection	**16b**	Cite studies that might appear to meet the inclusion criteria, but which were excluded, and explain why they were excluded.	Results 3.1, pp. 14-16, and Figure 2, p. 15. Full-text exclusion categories and counts are reported.
Study characteristics	**17**	Cite each included study and present its characteristics.	Results 3.2, pp. 16-18, especially Tables 5 and 6. Included study characteristics and synthesis roles are presented.
Risk of bias in studies	**18**	Present assessments of risk of bias for each included study.	Results 3.3, pp. 18-20, and Figures 3-4. Risk-of-bias results are presented for in vitro studies and the randomized controlled trial.
Results of individual studies	**19**	For all outcomes, present, for each study: (a) summary statistics for each group (where appropriate) and (b) an effect estimate	Results 3.4-3.5, pp. 20-25, and Figures 5-7. Individual study findings and forest-

Section and Topic	Item #	Checklist item	Location where item is reported
		and its precision (e.g. confidence/credible interval), ideally using structured tables or plots.	plot estimates are presented. Additional extracted data are available in supporting files.
Results of syntheses	20a	For each synthesis, briefly summarise the characteristics and risk of bias among contributing studies.	Results 3.2-3.5, pp. 16-25. Characteristics and risk of bias of contributing studies are summarized before and within the outcome syntheses.
Results of syntheses	20b	Present results of all statistical syntheses conducted. If meta-analysis was done, present for each the summary estimate and its precision (e.g. confidence/credible interval) and measures of statistical heterogeneity. If comparing groups, describe the direction of the effect.	Results 3.5, pp. 22-26; Figures 5-7; Table 7. Pooled mean differences, confidence intervals, p-values, and heterogeneity statistics are reported.
Results of syntheses	20c	Present results of all investigations of possible causes of heterogeneity among study results.	Results 3.5 and 3.7, pp. 22-28, and Discussion 4.5, pp. 31-32. Heterogeneity and influential comparisons are described.
Results of syntheses	20d	Present results of all sensitivity analyses conducted to assess the robustness of the synthesized results.	Results 3.6–3.7, pp. 16–19; Tables 8–9; Supplementary Table S5. Sensitivity, clustering, and leave-one-out analyses are reported.
Reporting biases	21	Present assessments of risk of bias due to missing results (arising from reporting biases) for each synthesis assessed.	Results 3.8, p. 28. Formal assessment was not performed because fewer than 10 comparisons were included per meta-analysis.
Certainty of evidence	22	Present assessments of certainty (or confidence) in the body of evidence for each outcome assessed.	Supporting Information, p. 39: Supplementary Table S2. Methods 2.8, p. 12, describes the GRADE approach.
**DISCUSSION**			
Discussion	23a	Provide a general interpretation of the results in the context of other evidence.	Discussion 4.1-4.4 and 4.7/4.11, pp. 29-33 and 37-38. General interpretation is provided in the context of previous reviews and included evidence.
Discussion	23b	Discuss any limitations of the evidence included in the review.	Discussion 4.5 and 4.9, pp. 31-35. Limitations of the evidence include heterogeneity, predominance of in vitro studies, and system/context dependence.
Discussion	23c	Discuss any limitations of the review processes used.	Discussion 4.9, p. 35. Limitations related to the review process, evidence base, outcome definitions, data availability, and synthesis assumptions are discussed.
Discussion	23d	Discuss implications of the results for practice, policy, and future research.	Discussion 4.8 and 4.10, pp. 34-36, and Conclusions, p. 38. Clinical implications and future research recommendations are discussed.
**OTHER INFORMATION**			
Registration and protocol	24a	Provide registration information for the review, including register name and registration number, or state that the review was not registered.	Methods 2.1, p. 7. Registration is reported in the Open Science Framework, with URL: https://osf.io/tsqf7.
Registration and protocol	24b	Indicate where the review protocol can be accessed, or state that a protocol was not prepared.	Methods 2.1, p. 7. The protocol location/access is provided through OSF.
Registration and protocol	24c	Describe and explain any amendments to information provided at registration or in the protocol.	Methods 2.1, p. 7. The manuscript states that no amendments to the registered protocol were made.
Support	25	Describe sources of financial or non-financial support for the review, and the role of the funders or sponsors in the review.	Funding statement, p. 39. The source of publication support is described.
Competing interests	26	Declare any competing interests of review authors.	Conflicts of Interest, p. 39. The authors declare no conflicts of interest.

Section and Topic	Item #	Checklist item	Location where item is reported
Availability of data, code and other materials	**27**	Report which of the following are publicly available and where they can be found: template data collection forms; data extracted from included studies; data used for all analyses; analytic code; any other materials used in the review.	Data Availability Statement and Supporting Information, p. 39; Methods 2.1, p. 7. Data/materials are included in the article/supporting files and the protocol is registered in OSF.

From: Page MJ, McKenzie JE, Bossuyt PM, Boutron I, Hoffmann TC, Mulrow CD, et al. The PRISMA 2020 statement: an updated guideline for reporting systematic reviews. BMJ 2021;372:n71. doi: 10.1136/bmj.n71. For more information, visit: http://www.prisma-statement.org/

Completion note: locations were mapped against the manuscript file supplied for submission. Page numbers refer to the rendered manuscript pages and may shift slightly if the manuscript layout is changed before resubmission.

Supplementary Table S2. Certainty of evidence according to study setting.

Outcome	Evidence setting	Evidence base	Main GRADE considerations	Certainty
Angular deviation	In vitro	6 studies; 7 comparisons	Methodological quality generally acceptable; variability in effect magnitude across systems and experimental conditions; pooled estimate remained significant and stable in sensitivity analyses.	Moderate ⨁⨁⨁◯
Coronal/platform deviation	In vitro	5 studies; 6 comparisons	Methodological quality generally acceptable; variability across systems and experimental conditions; pooled confidence interval included no difference between guide designs.	Low ⨁⨁◯◯
Apical deviation	In vitro	5 studies; 6 comparisons	Methodological quality generally acceptable; variability in effect magnitude across systems and site conditions; direction of the pooled estimate remained consistent across sensitivity analyses, including analyses addressing potential within-cluster dependence.	Moderate ⨁⨁⨁◯
Angular deviation	Clinical	1 randomized controlled trial	Direct clinical evidence; RoB 2 assessment indicated some concerns; evidence limited to a single randomized trial.	Low ⨁⨁◯◯
Coronal/platform deviation	Clinical	1 randomized controlled trial	Direct clinical evidence; RoB 2 assessment indicated some concerns; evidence limited to a single randomized trial.	Low ⨁⨁◯◯
Apical deviation	Clinical	No directly comparable clinical evidence	No directly comparable clinical evidence available.	Not rated

Note: Certainty was assessed separately by study setting using an adapted GRADE approach. For in-vitro evidence, certainty refers to comparative technical accuracy under controlled experimental conditions and does not imply equivalent certainty of clinical effectiveness. QUIN informed the assessment of the in-vitro evidence, whereas RoB 2 informed the assessment of the randomized clinical trial.

Supplementary Table S3. Full-text reports excluded after eligibility assessment and reasons for exclusion.

No.	Study	Title	Reason for exclusion
1	Shi et al. (2023)	A systematic review of the accuracy of digital surgical guides for dental implantation.	Review/scoping/systematic review
2	Patel et al. (2026)	Accuracy of handpiece-guided implant surgical systems: A scoping review.	Review/scoping/systematic review
3	Tallarico et al. (2021)	Accuracy of surgical templates with and without metallic sleeves in case of partial arch restorations: A systematic review.	Review/scoping/systematic review
4	Esperouz et al. (2025)	Accuracy of guided implant placement using surgical guides with or without metal sleeves: Systematic review and meta-analysis.	Review/scoping/systematic review
5	Gargallo-Albiol et al. (2019)	Advantages and disadvantages of implant navigation surgery: a systematic review.	Review/scoping/systematic review
6	Chen et al. (2024)	Accuracy of open-sleeved vs. closed-sleeved static computer-assisted implant systems in immediate maxillary molar implant placement: An in vitro study.	No direct sleeve-free comparison
7	Guentsch et al. (2022)	Precision and trueness of computer-assisted implant placement using static surgical guides with open and closed sleeves: An in vitro analysis.	No direct sleeve-free comparison
8	Li et al. (2022)	Open-sleeve templates for computer-assisted implant surgery at healed or extraction sockets: An in vitro comparison to closed-sleeve guided system and free-hand approach.	No direct sleeve-free comparison
9	Dusmukhamedov et al. (2022)	Effect of Anchor-Guiding Sleeve Length on the Accuracy of Computer-Guided Flapless Implant Surgery.	No direct sleeve-free comparison
10	Guentsch et al. (2021)	Precision and trueness of implant placement with and without static surgical guides: An in vitro study.	No direct sleeve-free comparison
11	Schnutenhaus et al. (2021)	Does the macro design of an implant affect the accuracy of template-guided implantation? A prospective clinical study.	No direct sleeve-free comparison
12	Schnutenhaus et al. (2024)	Influence of the macrodesign of an implant and the sleeve system on the accuracy of template-guided implant placement: A prospective clinical study.	No direct sleeve-free comparison
13	El Kholy et al. (2019)	The influence of guided sleeve height, drilling distance, and drilling key length on the accuracy of static Computer-Assisted Implant Surgery.	No direct sleeve-free comparison
14	Liang et al. (2019)	In Vitro Experimental Study of the Effect of Adjusting the Guide Sleeve Height and Using a Visual Direction-Indicating Guide on Implantation Accuracy.	No direct sleeve-free comparison
15	Meitner et al. (2022)	Evaluation of the Accuracy of a New Geometric Approach to Implant Guidance.	No direct sleeve-free comparison
16	Laederach et al. (2017)	Deviations of different systems for guided implant surgery.	No direct sleeve-free comparison

No.	Study	Title	Reason for exclusion
17	Mo et al. (2023)	Accuracy of a 3D printed sleeveless guide system used for fiber post removal: An in vitro study.	Not implant placement
18	Martinho et al. (2023)	A Cadaver-based Comparison of Sleeve-guided Implant-drill and Dynamic Navigation Osteotomy and Root-end Resections.	Not implant placement
19	de Mello Vasconcellos et al. (2025)	Modified Surgical Guide for Root Sectioning in the Socket Shield Technique-In Vitro Study.	Not implant placement
20	Chen et al. (2026)	Analysis of Factors Affecting the Accuracy of Static-guided Endodontics.	Not implant placement
21	Uzunov et al. (2025)	Incorporating Irrigation Channels in Sleeved Surgical Guides.	Technical/manufacturing only
22	Lo Russo et al. (2023)	Surgical guides for dental implants: Measurement of the accuracy using a freeware metrology software program.	Technical/manufacturing only
23	Lo Russo et al. (2023)	Effect of Fabrication Technology on the Accuracy of Surgical Guides for Dental-Implant Surgery.	Technical/manufacturing only
24	Lim et al. (2022)	Sleeve insert scan body to predict implant placement position by using implant surgical guides: A dental technique.	Technical/manufacturing only
25	Orgev et al. (2021)	Addition of an irrigation channel to a surgical template to facilitate cooling during implant osteotomy.	Technical/manufacturing only
26	Seidel et al. (2025)	Accuracy of Drill Sleeve Housing in 3D-Printed and Milled Implant Surgical Guides: A 3D Analysis Considering Machine Type, Layer Thickness, Sleeve Position, and Support Type.	Technical/manufacturing only
27	Oh et al. (2019)	Assessment of metal sleeve-free 3D-printed implant surgical guides.	No extractable data
28	Shah et al. (2022)	An evaluation of virtually planned and 3D-printed stereolithographic surgical guides from CBCT and digital scans: An in vitro study.	No extractable data
29	Kernen et al. (2016)	Accuracy of Three-Dimensional Printed Templates for Guided Implant Placement Based on Matching a Surface Scan with CBCT.	No extractable data
30	Matta et al. (2017)	The impact of the fabrication method on the three-dimensional accuracy of an implant surgery template.	No extractable data
31	Pieralli et al. (2020)	How Accurate Is Oral Implant Installation Using Surgical Guides Printed from a Degradable and Steam-Sterilized Biopolymer?	No extractable data
32	Schnutenhaus et al. (2018)	Precision of sleeveless 3D drill guides for insertion of one-piece ceramic implants: a prospective clinical trial.	Case/non-comparative
33	Burkhardt et al. (2020)	Guided implant surgery for one-piece ceramic implants: a digital workflow.	Case/non-comparative
34	Tallarico et al. (2019)	Accuracy of newly developed sleeve-designed templates for insertion of dental implants: A prospective multicenters clinical trial.	Case/non-comparative

No.	Study	Title	Reason for exclusion
35	Simpson et al. (2020)	Computer-Guided Surgery Using Human Allogenic Bone Ring With Simultaneous Implant Placement: A Case Report.	Case/non-comparative
36	Abouzeid et al. (2024)	A modified protocol merging two published techniques for computer guided zygomatic implants surgery: a technical note.	Case/non-comparative

Supplementary Table S4. Statistical unit and availability of information relevant to within-cluster dependence in studies contributing to quantitative synthesis.

Study	Higher-level experimental unit	Mean observations per unit (m)	Multiple observations per unit?	Primary analysis explicitly accounted for dependence?	ICC/design effect or directly usable cluster-adjusted variance reported?
Chung et al.	Mandibular model	2	✓	No explicit within-model adjustment identified	✗
Sittikornpaiboon et al.	3D-printed model	2	✓	No explicit within-model adjustment identified	✗
Dulla et al.*	Maxillary model	≈2	✓	No explicit within-model adjustment identified	✗
Galli et al.	Maxillary cast	3 (sockets) / 4 (healed)	✓	Yes — linear mixed model	✗
Raabe et al.	Maxillary model	≈2	✓	No explicit within-model adjustment identified	✗
Ballesteros et al.*	Printed maxillary model	6	✓	No explicit within-model adjustment identified	✗
Hang and Guentsch	Mandibular replica	1	No	N/A — one implant per replica	N/A
Tallarico et al.	Patient	2.73 (sleeved) / 3.27 (sleeve-free)	✓	No explicit patient-level clustering adjustment identified	✗
Aboul Azayem et al.	Printed mandibular model	2	✓	No explicit within-model adjustment identified	✗

*Note. The table summarises information relevant to unit-of-analysis assessment according to Cochrane principles [37]. ✓, present/applicable; ✗, not reported and not derivable from the published report; N/A, not applicable. "No explicit adjustment identified" refers specifically to the statistical methods reported in the publication and does not imply that an unreported procedure was not undertaken. This table is descriptive and does not constitute an additional risk-of-bias or study-quality assessment. *Sensitivity-analysis contribution.*

Supplementary Table S5. Sensitivity of pooled estimates to assumed within-cluster correlation.

Panel A. Primary meta-analytic structure with clustering adjustment.

Outcome	ICC	Comparisons	Mean difference	95% CI	p-value	I^2 (%)
Angular	0.00	8	-0.613	-1.123 to -0.103	0.019	81.7
	0.10	8	-0.606	-1.109 to -0.102	0.018	77.5
	0.25	8	-0.594	-1.086 to -0.102	0.018	71.6
	0.50	8	-0.571	-1.040 to -0.103	0.017	62.7
	0.75	8	-0.546	-0.988 to -0.105	0.015	54.5
	1.00	8	-0.523	-0.937 to -0.108	0.013	46.8
Coronal/platform	0.00	7	-0.027	-0.162 to 0.107	0.689	81.6
	0.10	7	-0.032	-0.161 to 0.098	0.633	78.7
	0.25	7	-0.038	-0.160 to 0.085	0.545	74.6
	0.50	7	-0.048	-0.159 to 0.064	0.400	67.9
	0.75	7	-0.056	-0.158 to 0.046	0.282	61.3
	1.00	7	-0.062	-0.156 to 0.032	0.199	54.8
Apical	0.00	6	-0.274	-0.499 to -0.048	0.017	90.0
	0.10	6	-0.269	-0.493 to -0.045	0.019	87.8
	0.25	6	-0.262	-0.484 to -0.039	0.021	84.7

Outcome	ICC	Comparisons	Mean difference	95% CI	p-value	I² (%)
	0.50	6	-0.250	-0.468 to -0.031	0.025	80.2
	0.75	6	-0.237	-0.450 to -0.024	0.029	76.0
	1.00	6	-0.225	-0.432 to -0.017	0.034	72.2

Panel B. Galli site conditions combined before clustering adjustment.

Outcome	ICC	Comparisons	Mean difference	95% CI	p-value	I² (%)
Angular	0.00	7	-0.511	-0.934 to -0.089	0.018	72.9
	0.10	7	-0.488	-0.881 to -0.094	0.015	63.9
	0.25	7	-0.455	-0.813 to -0.097	0.013	53.7
	0.50	7	-0.426	-0.760 to -0.092	0.013	40.9
	0.75	7	-0.417	-0.748 to -0.086	0.014	30.8
	1.00	7	-0.416	-0.750 to -0.082	0.015	22.1
Coronal/platform	0.00	6	-0.045	-0.154 to 0.065	0.426	79.3
	0.10	6	-0.048	-0.156 to 0.060	0.382	75.2
	0.25	6	-0.053	-0.159 to 0.053	0.325	69.9
	0.50	6	-0.060	-0.162 to 0.043	0.252	62.0
	0.75	6	-0.064	-0.163 to 0.034	0.201	54.4

Outcome	ICC	Comparisons	Mean difference	95% CI	p-value	I^2 (%)
	1.00	6	-0.067	-0.162 to 0.027	0.162	47.0
Apical	0.00	5	-0.223	-0.419 to -0.028	0.025	86.9
	0.10	5	-0.211	-0.398 to -0.023	0.028	82.9
	0.25	5	-0.193	-0.369 to -0.016	0.032	78.4
	0.50	5	-0.170	-0.336 to -0.004	0.044	72.9
	0.75	5	-0.157	-0.318 to 0.005	0.057	68.6
	1.00	5	-0.149	-0.309 to 0.012	0.070	64.9

Note. Mean differences were calculated as sleeve-free minus metallic-sleeved; negative values favour sleeve-free guides. Sampling variances were adjusted using DE = 1 + (m − 1)ρ. Mean differences were unchanged by the adjustment; increasing ρ increased sampling variance and reduced study weight. ICC = 1.00 represents a deliberately conservative maximal-dependence assumption rather than an empirically estimated correlation. In Panel B, the healed- and extraction-site data from Galli et al. were combined before applying the clustering adjustment.
